# A synopsis of the expanded *Rhaphiolepis* (Maleae, Rosaceae)

**DOI:** 10.3897/phytokeys.154.52790

**Published:** 2020-08-03

**Authors:** Bin-Bin Liu, Yu-Bing Wang, De-Yuan Hong, Jun Wen

**Affiliations:** 1 State Key Laboratory of Systematic and Evolutionary Botany, Institute of Botany, Chinese Academy of Sciences, Beijing 100093, China Chinese Academy of Sciences Beijing China; 2 Department of Botany, National Museum of Natural History, Smithsonian Institution, PO Box 37012, Washington, DC 20013-7012, USA National Museum of Natural History Washington United States of America; 3 Key Laboratory of Three Gorges Regional Plant Genetics & Germplasm Enhancement (CTGU)/Biotechnology Research Center, China Three Gorges University, Yichang, 443002, China China Three Gorges University Yichang China

**Keywords:** *Eriobotrya*, lectotype, new name, nomenclature, taxonomy, typification, validation

## Abstract

As part of the integrative systematic studies on the tribe Maleae, a synopsis of the expanded *Rhaphiolepis* is presented, recognizing 45 species. Three new forms were validated: R.bengalensisf.contracta B.B.Liu & J.Wen, R.bengalensisf.intermedia B.B.Liu & J.Wen, and R.bengalensisf.multinervata B.B.Liu & J.Wen, and four new combinations are made here: R.bengalensisf.angustifolia (Cardot) B.B.Liu & J.Wen, R.bengalensisf.gigantea (J.E.Vidal) B.B.Liu & J.Wen, *R.laoshanica* (W.B.Liao, Q.Fan & S.F.Chen) B.B.Liu & J.Wen, and *R.latifolia* (Hook.f.) B.B.Liu & J.Wen. Furthermore, one new name, *Rhaphiolepisyui* B.B.Liu & J.Wen is proposed here, and three taxa were reduced as new synonyms. We also provide lectotypification for 13 names: *Crataegusbibas*, *Eriobotryaphilippinensis*, *Mespilusspiralis*, *Opaintegerrima*, *Photinialuzonensis*, *Rhaphiolepisbrevipetiolata*, R.ferrugineavar.serrata, *R.fragrans*, *R.gracilis*, *R.hainanensis*, *R.kerrii*, R.indica subsp. umbellatavar.liukiuensis, and *R.parvibracteolata*.

## Introduction

The three-subfamily classification system of Rosaceae, Amygdaloideae, Dryadoideae, and Rosoideae, has been accepted and stabilized with a series of molecular phylogenetic studies (Morgan et al. 1994; Potter et al. 2007; Xiang et al. 2017; Zhang et al. 2017). As one of the nine tribes in Amygdaloideae, the apple tribe Maleae consists of ca. 1000 species widely distributed throughout the Northern Hemisphere (Phipps et al. 1990). Some members in Maleae have shown great economic importance, especially as fruits, e.g. apples (*Malusdomestica* (Suckow) Borkh.), pears (*Pyruscommunis* L.), and loquats (*Rhaphiolepisbibas* (Lour.) Galasso & Banfi), as well as some ornamentals, e.g. chokeberries (*Aronia* Medik.), cotoneasters (*Cotoneaster* Medik.), firethorns (*Pyracantha* M.Roem.), hawthorns (*Crataegus* L.), photinias (*Photinia* Lindl.), rowans (*Sorbus* L.), and serviceberries (*Amelanchier* Medik.). Members of Maleae, therefore, have attracted the attention of many horticulturists, pomologists, and taxonomists.

A recent phylogenomic analysis of *Rhaphiolepis* Lindl. and *Eriobotrya* Lindl. in the framework of Maleae (Rosaceae) strongly supported the paraphyly of *Eriobotrya*, with *Rhaphiolepis* nested within it (Liu et al. 2020b). These two genera were thus considered to be congeneric and treated as *Rhaphiolepis*, which has the nomenclatural priority. The expanded *Rhaphiolepis* has two synapomorphies: the proportionally large seed with rounded or wide-elliptic cross-section and the absence of endosperm (Aldasoro et al. 2005; Liu et al. 2020b). Furthermore, frequent hybridizations may have occurred in the diversification of the expanded *Rhaphiolepis*, which may explain some of the reported topological incongruences between nuclear and chloroplast DNA data within the genus (Liu et al. 2020b). Hybridization events have been reported in several lineages of the apple tribe, such as *Micromeles* Decne., *Pseudocydonia* (C.K.Schneid.) C.K.Schneid. (Lo and Donoghue 2012), and *Phippsiomeles* B.B.Liu & J.Wen (Liu et al. 2019), as well as the *Amelanchier*-*Malacomeles* (Decne.) Decne.-*Peraphyllum* Nutt. clade (Liu et al. 2020a). It is also very common in many lineages of angiosperms, e.g. Gesneriaceae (Kleinkopf et al. 2019), Magnoliaceae (Wang et al. 2020), Vitaceae (Wen et al. 2018, 2020), and Wightiaceae (Xia et al. 2019).

Most of the species of the expanded *Rhaphiolepis* are trees and shrubs, distributed from subtropical East Asia to tropical Southeast Asia. The loquat species (*Rhaphiolepisbibas* = *Eriobotryajaponica* (Thunb.) Lindl.) belongs to this genus, and has been widely cultivated all over the world as fruits and ornamentals. Furthermore, several other species, such as *Rhaphiolepisindica* (L.) Lindl. and *R.umbellata* (Thunb.) Makino have been used as ornamentals with their abundant white flowers and persistent red fruits. Thunberg (1784) described the first species, i.e. the loquat, as *Mespilusjaponica* Thunb., and since then nearly 242 names have been published in the last two centuries. Previous studies were primarily regional in scope along with floristic treatments, e.g., Flora of British India (Hooker 1878), Flora of Japan (Ohwi 1965), Flore du Cambodge, du Laos et du Vietnam (Vidal 1968), Flora of Thailand (Vidal 1970), Flora of Taiwan (Ohashi 1993), and Flora of China (Kuan and Yu 1974; Gu and Spongberg 2003). While these names have never been well evaluated comprehensively, and some of them have been largely neglected since their description, such as *Rhaphiolepiscrataegoides* M.Roem. and *R.laevis* Lodd. ex G.Don. Liu et al. (2020c) provided typifications for 23 names related to *Eriobotrya*. We herein evaluate all names published previously and provide a genus-wide synopsis of *Rhaphiolepis*, while the horticultural cultivars will not be in the scope of this study, such as Eriobotryajaponicaf.variegata and × *Rhaphiobotrya* ‘Coppertone’ (Coombes and Robertson 2008). We hope the taxonomic framework presented here will stimulate in-depth evolutionary studies of this widely distributed Asian lineage of Maleae using collections-based tools (Wen et al. 2017; Funk 2018).

## Materials and methods

We reviewed all names published previously, by checking all available online resources, such as Tropicos (2020: https://www.tropicos.org), IPNI (2020: https://www.ipni.org/), and the Plant List (2013: http://www.theplantlist.org/), furthermore, all the regional floras (e.g. Hooker 1878; Ohwi 1965; Vidal 1968, 1970; Ohashi 1993; Gu and Spongberg 2003) and related original literature of each taxon. We have followed the most recent taxonomic treatments in the regional floras, such as Flora of China (Gu and Spongberg 2003) and Flore du Cambodge, du Laos et du Vietnam (Vidal 1968), and the unresolved names were recognized temporarily herein. The rules governing the holotype and lectotype followed McNeill (2014), Turland et al. (2018), and Turland (2019). Thanks to the rapid digitization of plant specimens around the world, we checked the type specimens via JSTOR (2020) or personal communications with the herbaria. A total of 184 images of type specimens have been evaluated, and these specimens are from the following herbaria, A, B, BM, C, E, HBG, K, L, M, MO, MSC, NY, P, TCD, U, UPS, CNMN, and WU. We also visited some herbaria in China and USA for the type material, CDBI, HITBC, IBK, IBSC, KUN, PE, SN, SYS, SZ, US, and WUK. The herbarium code followed Index Herbariorum (2020).

## Taxonomy

### Rhaphiolepis

Taxon classificationPlantaeRosalesRosaceae

Lindl. in Bot. Reg.: ad t. 468. 1 Jul 1820 (‘ Raphiolepis’) (nom. & orth. cons.).

5822E933-E715-5F18-9C4B-3B41CAF3E44C

= Eriobotrya Lindl., Trans. Linn. Soc. London 13: 96, 102. 1821. Type: Eriobotryajaponica (Thunb.) Lindl. ≡ Mespilusjaponica Thunb. (= Rhaphiolepisbibas (Lour.) Galasso & Banfi).
= Opa Lour., Fl. Cochinch.: 304, 308. Sep 1790. Type (vide McVaugh 1956): Opametrosideros Lour. (= Rhaphiolepisindica (L.) Lindl.).
=× Rhaphiobotrya Coombes, Plantsman n.s., 7(3): 164. 2008 (Eriobotrya Lindl. × Rhaphiolepis Lindl.).

#### Type.

*Rhaphiolepisindica* (L.) Lindl.≡*Crataegusindica* L.

#### Description.

***Trees, small trees, or shrubs***, 40–100[–200] dm. ***Stems*** ca. 1, erect; bark gray-brown; short shoots absent; unarmed; hairy. ***Leaves*** persistent, cauline, simple; stipules deciduous or ± persistent, free, on the extreme base of petiole, rarely intrapetiolarly connate, subulate, caducous, or subulate, small, margin entire; petiole present; blade ± elliptic to oblong-lanceolate, 2–40 cm, leathery, margins flat or reflex, serrate, dentate or entire, venation penninerved (craspedodromous or camptodromous). ***Inflorescences*** in terminal racemes, panicles, or compound racemes, many-flowered. ***Pedicels*** present, short, or nearly absent. ***Flowers***: perianth and androecium epigynous, 15–20 mm diam.; hypanthium campanulate, cupular, tubular, or obconical, the free part inside lined with an intrastaminal disk, open at the top; sepals 5, persistent or caducous; petals 5, white, yellow, or pink, obovate or orbicular, base clawed; stamens 15–20(-40); ovary inferior, carpels 2–5, ventrally and laterally connate (in upper part ventrally free), or completely connate with each other and dorsally adnate to the hypanthium, the hairy apex exposed; ovules normally 2 per carpel, rarely more; styles 2–5, connate at base and often pubescent; stigma truncate. ***Fruits*** a pome, yellowish, yellowish red, brown, dark purplish-brown, bluish, or purplish-black, subglobose, globose, or obovate, fleshy or dry, flesh mostly of hypanthial origin, sclereids absent or present, endocarp (core) thin, membranous. ***Seeds*** 1–3, large, with a thin but firm testa; endosperm absent, cotyledons thick. 2*n* = 34.

About 45 species (Vidal 1965, 1968, 1970; Kalkman 1973, 2004; Gu and Spongberg 2003) in East & Southeast Asia and the Himalayas, south to Borneo and Sumatra.

### 1. Rhaphiolepis angustissima

Taxon classificationPlantaeRosalesRosaceae

(Hook.f.) B.B.Liu & J.Wen, Front. Plant Sci. 10-1731: 10. 2020.

5EE64D06-3769-5C1B-AE03-8F42E00E09DF

≡ Eriobotryaangustissima Hook.f., Fl. Brit. India [J. D. Hooker] 2(5): 372. 1878. Type: India. Khasia. alt. 5000 ft., without date, *J.D. Hooker & T. Thomson s.n.* (lectotype, designated by Vidal 1965, pg. 574: K [barcode K000758406]! “type”; isolectotype: BM [barcode BM000602192]!, “isotype”).
≡ Pyrusangustissima (Hook.f.) M.F.Fay & Christenh., Global Fl. 4: 95. 2018. Type: Based on Eriobotryaangustissima.

#### Distribution.

India (Mt. Khasia) and Vietnam.

### 2. Rhaphiolepis balgooyi

Taxon classificationPlantaeRosalesRosaceae

(K.M.Wong & Ent) B.B.Liu & J.Wen, Front. Plant Sci. 10-1731: 10. 2020.

4F17AB56-7A97-5BE5-AA4D-1C6F6F3266EA

≡ Eriobotryabalgooyi K.M.Wong & Ent, Pl. Ecol. Evol. 147(1): 136. 2014. Type. Malaysia. Sabah, Ranau District, Bukit Babi [Pig Hill] on the south-east side of Mount Kinabalu, 6°03'N, 116°36'E, 2000–2300 m, 25 May 1984, *J.H. Beaman et al. 9871* (holotype: K [barcode K000618095]!; isotype: MSC).

#### Distribution.

Malaysia (Borneo on Mt. Kinabalu and Mt. Tambuyukon).

### 3. Rhaphiolepis bengalensis

Taxon classificationPlantaeRosalesRosaceae

(Roxb.) B.B.Liu & J.Wen, Front. Plant Sci. 10-1731: 10. 2020.

6E576089-4B28-5D5B-A597-F58F7013FCA6

≡ Mespilusbengalensis Roxb., Fl. Ind. (ed. 1832) 2: 510. 1832. Type: India. 1824, *N. Wallich 668.2* (neotype, designated by Vidal 1965, pg. 567: K [barcode K001111550]!, “lectotype”; isoneotype: P [barcode P02143255]!), “isolectotype”. cf. note in Liu et al. 2020c, pg. 113).
≡ Eriobotryabengalensis Hook.f., Fl. Brit. India [J. D. Hooker] 2(5): 371. 1878. Type: Based on Mespilusbengalensis.

### 3a. Rhaphiolepis bengalensis f. bengalensis

Taxon classificationPlantaeRosalesRosaceae

1B81C319-ED9D-52A9-B674-45A667DDFDF6

= Alsodeiagrandis Miq., Fl. Ned. Ind., Eerste Bijv. 3: 391. 1861. Type: Indonesia. “Sumatra orient. in regionibus interioribus prov. Palembang, prope Muara-enim”, *s.coll. HB4023* (holotype: U [barcode U0005827]!).
= Eriobotryatinctoria Kurz, Prelim. Rep. For. Veg. Pegu, App. B. 48. 1875, in clavi. Type: not designated.

#### Distribution.

widely distributed from East Himalaya (Sikkim and Assam) through Bangladesh (Chittagong) to Myanmar, Laos, Cambodia, Vietnam, Malay Peninsula, Sumatra, and Borneo.

### 3b. Rhaphiolepis bengalensis (Roxb.) B.B.Liu & J.Wen f. angustifolia

Taxon classificationPlantaeRosalesRosaceae

(Cardot) B.B.Liu & J.Wen comb. nov.

BE541043-4ED4-5F1C-9916-D3E89E2BB10F

urn:lsid:ipni.org:names:77210691-1

≡ Eriobotryabengalensis(Roxb.)Hook.f.var.angustifolia Cardot, Notul. Syst. (Paris) 3: 371. 1918. Type: China. Yunnan: Hay-y près Lou-Lan, Pau Ngueou, 29 March 1907, *F. Ducloux 4719* (lectotype, designated by Liu et al. 2020c, pg. 103: P [barcode P02143256]!; isolectotype: P [barcode P02143257]!).
≡ Eriobotryabengalensis(Roxb.)Hook.f.f.angustifolia (Cardot) J.E.Vidal, Adansonia, n.s. 5: 569. 1965. Type: Based on Eriobotryabengalensisvar.angustifolia.

#### Distribution.

China (Yunnan).

### 3c. Rhaphiolepis bengalensis (Roxb.) B.B.Liu & J.Wen f. contracta

Taxon classificationPlantaeRosalesRosaceae

B.B.Liu & J.Wen f. nov.

9D37FDEB-E6FE-57BF-AFA8-F22FF80C0C30

urn:lsid:ipni.org:names:77210693-1

≡ Eriobotryabengalensis(Roxb.)Hook.f.f.contracta J.E.Vidal, Adansonia, n.s. 5: 569. 1965, *nom. inval.* Type: Vietnam. Annam: sommet du Nui Bach Ma, Station d’altitude un peu au Sud de Huê Alt. 1400–1500 m, Le 6 September 1938, *E. Poilane 27620* (holotype: P [barcode P03650248]!, isotype: P [barcode P03650249]!). Annam: Nui Bach Ma Station d’altitude de Huê 1400–1500 m, d’alt. Lé 12 December 1940, *E. Poilane 31104* (paratypes: P [barcode P03650258, P03650259]!). Bachma (Centre-Vietnam), 23 August 1943, *J.E. Vidal 36* (paratype: P [barcode P03650257]!). Annam: Col des nuages près Tourane Forêt 900 m, d’altitude Le 14 September 1923, *E. Poilane 7986* (paratypes: P [barcode P03650251, P03650253]!). Prov. Quang Nam: *E. Poilane 11* (syntype). Annam: Massif du Ngok Guga près de Dakto prov. du Kontum Le 25 February 1946, alt. 1000 m, *E. Poilane 35584* (paratypes: P [barcode P03650240, P03650241)!). S. Annam: massif du Hon Ba, 31 August 1918, *A. Chevalier 38718* (paratype: P [barcode P03650239]!). Sud. Annam: Prov. Nha Trang: Massif du Hon Ba, 1000–1100 m alt., 4 September 1918, *A. Chevalier 38832* (paratypes: P [barcode P03650246, P03650247]!). Prov. Nha Trang: Massif du Hon Ba, 1000–1500 m alt., 4 September 1918, *A. Chevalier 38892* (paratypes: P [barcode P03650233, P03650238, P03650245]). [Note A]

#### Distribution.

Vietnam.

#### Note A.

Rhaphiolepisbengalensis(Roxb.)B.B.Liu & J.Wenf.contracta B.B.Liu & J.Wen, f. nov.^**^Vidal (1965) cited nine collections as syntypes in the protologue, but he did not indicate a single type. Eriobotryabengalensis(Roxb.)Hook.f.f.multinervata J.E.Vidal was thus invalidly published (Art. 40.1: Turland et al. 2018). We validated Rhaphiolepisbengalensisf.multinervata as a new form by reference to designating one duplicate (P03650248) of the first collections cited by Vidal (1965) as the holotype, and the diagnosis followed Vidal (1965).

### 3d. Rhaphiolepis bengalensis (Roxb.) B.B.Liu & J.Wen f. gigantea

Taxon classificationPlantaeRosalesRosaceae

(J.E.Vidal) B.B.Liu & J.Wen comb. nov.

30CF448A-0EB8-5DFE-A7A8-0A7CB925A6A6

urn:lsid:ipni.org:names:77210695-1

≡ Eriobotryabengalensis(Roxb.)Hook.f.f.gigantea J.E.Vidal, Adansonia, n.s. 5: 569. 1965. Type: Myanmar. *Parkinson 314* (holotype: K)

#### Distribution.

Myanmar.

### 3e. Rhaphiolepis bengalensis (Roxb.) B.B.Liu & J.Wen f. intermedia

Taxon classificationPlantaeRosalesRosaceae

B.B.Liu & J.Wen f. nov.

F11C2C31-2181-59AD-A284-A2B441ABC814

urn:lsid:ipni.org:names:77210697-1

≡ Eriobotryabengalensis(Roxb.)Hook.f.f.intermedia J.E.Vidal, Adansonia, n.s. 5: 568. 1965, *nom. inval.* Type: Myanmar. “In thicket on the western flank of the N’Maikha-Salween divide, east of Hpimaw. Lat. 26°N, alt. 10000 feet. East Upper Burmah”, April 1919, *G. Forrest 17845* (holotype: E [barcode E00072976]!; isotypes: E [barcode E00072977]!, K). Région de Huê, “Bachma, Centre Vietnam, 1200 m”, 21 January 1944, *J.E. Vidal 35A* (paratype: P [barcode P03650235]!); “Km. 13, Route de Bachma, Centre Vietnam, 1200 m”, 12 March 1944, *J.E. Vidal 35B* (paratype: P [barcode P03650234]!); “Km. 12.5 Route de Bachma Centre Vietnam”, 6 April 1944, *J.E. Vidal 35C* (paratype: P [barcode P03650231]!). [Note B]

#### Diagnosis.

A forma typica differt stylis 4 frequentioribus (Vidal 1965).

#### Distribution.

Myanmar.

#### Note B.

Four gatherings were cited in the protologue by Vidal (1965), but none of them was not designated as type. Eriobotryabengalensisf.intermedia was thus invalidly published (Art. 40.1: Turland et al. 2018) despite the lectotypification designated by Liu et al. (2020c) for this name. We validated Rhaphiolepisbengalensisf.intermedia as a new form by reference to designating one duplicate (E00072976) of the first gathering cited by Vidal (1965) as the holotype, and the diagnosis followed Vidal (1965).

### 3f. Rhaphiolepis bengalensis (Roxb.) B.B.Liu & J.Wen f. multinervata

Taxon classificationPlantaeRosalesRosaceae

B.B.Liu & J.Wen f. nov.

4760D7FC-C5B3-5364-A88F-3444E1409104

urn:lsid:ipni.org:names:77210699-1

≡ Eriobotryabengalensis(Roxb.)Hook.f.f.multinervata J.E.Vidal, Adansonia, n.s. 5: 569. 1965, *nom. inval.* Type: Thailand. Siam, Chiang Mai, Doi Angka (now as Doi Inthanon), ca. 1400 m, 16 July 1922, *A.F.G. Kerr 6293* (holotype: P [barcode P03650228]!). Siam: Doi Pa Kao, ca. 1200 m, 7 May 1921, *A.F.G. Kerr 5372* (paratype: P [barcode P03650229]!). 1964, *B. Hansen et al. 10797* (paratypes: C, P [barcode P03650232]!). Siam. Kanchanaburi: Si Sawat, ca. 600 m, 17 January 1926, *A.F.G. Kerr 10235* (paratypes: K, P [barcode P03650230]!). [Note C]

#### Distribution.

Thailand.

#### Diagnosis.

A forma typica differt lamina venis utrinque 15–20 (Vidal 1965).

#### Note C.

Eriobotryabengalensisf.multinervata was invalidly published (Art. 40.1: Turland et al. 2018), because four gatherings were cited in the protologue by Vidal (1965), *A.F.G. Kerr 6293*, *A.F.G. Kerr 5372*, *A.F.G. Kerr 10235*, and *B. Hansen et al. 10797*. We herein validated Rhaphiolepisbengalensisf.multinervata as a new form by reference to designating *A.F.G. Kerr 6293* (P03650228) as the holotype which was in a better condition, and the diagnosis followed Vidal (1965).

### 4. Rhaphiolepis bibas

Taxon classificationPlantaeRosalesRosaceae

(Lour.) Galasso & Banfi, Ital. Botanist 9: 66. 2020.

3D0CBCB4-3938-5599-BCF7-F140FE40852F

≡ Crataegusbibas Lour., Fl. Cochinch. 1: 319. 1790. Type: Plukenet, L. 1705. Amaltheumbotanicum pag. 26. tab. 371. fig. 2. (**lectotype, designated here**). [Note D]
≡ Pyrusbibas (Lour.) M.F.Fay & Christenh., Global Fl. 4: 98. 2018. Type: Based on Crataegusbibas.
= Mespilusjaponica Thunb., Fl. Jap. (Thunberg) 206. 1784. Type: Japan. *Thunberg* s.n. (holotype: UPS-THUNB accession no. 11908).
≡ Eriobotryajaponica (Thunb.) Lindl., Trans. Linn. Soc. London 13: 102. 1821. Type: Based on Mespilusjaponica.
≡ Photiniajaponica (Thunb.) Benth. & Hook.f. ex Asch. & Schweinf., Mém. Inst. Égypt. [Illustr. Fl. Egypt.]. Type: Based on Mespilusjaponica.
≡ Rhaphiolepisloquata B.B.Liu & J.Wen, Front. Plant Sci. 10-1731: 11. 2020. nom. illeg. Type: Based on Mespilusjaponica.

#### Distribution.

Native in Chongqing (Nanchuan) and Hubei (Yichang) of China. As an economically important fruit, this species has been widely cultivated in central & south China, as well as in Japan, Korea, India, and some countries in Southeast Asia.

#### Note D.

**Figure 1.**
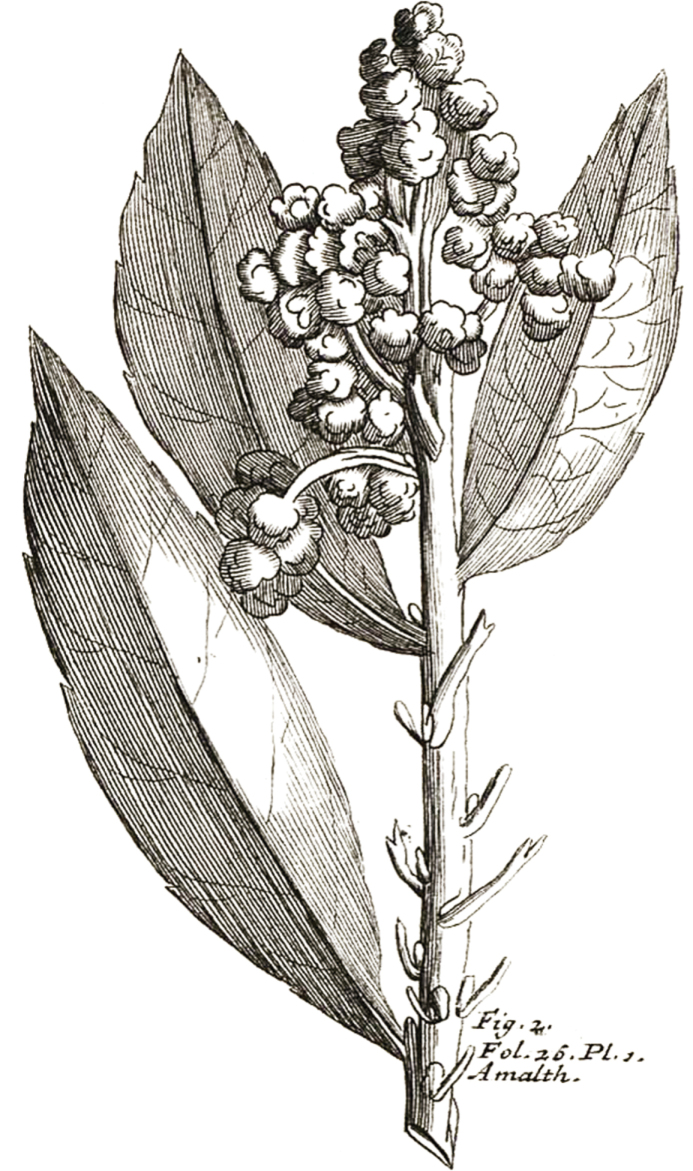
Lectotype of *Crataegusbibas*.

Loureiro (1790) described *Crataegusbibas* Lour. in his “Flora Cochinchinensis” and cited one illustration (Fig. 1) published in Plukenet’s book “Amaltheumbotanicum” in the protologue. This is because Loureiro had his collections of specimens that may contain (or may have contained) specimens of this species. This illustration is thus designated as the lectotype of *C.bibas* herein.

### 5. Rhaphiolepis brevipetiolata

Taxon classificationPlantaeRosalesRosaceae

J.E.Vidal, Fl. Cambodge, Laos & Vietnam Fasc. 6, 88. 1968, in adnot.

659596FF-A4A8-5A4A-A01B-A8ED07665952

#### Type.

Vietnam. “Prov. de Khanh Hoa (Nha Trang): région de Nha Trang, 1600 m”, 19 May 1922, *E. Poilane 3464* (**lectotype, designated here**: P [barcode P03206033]; isolectotype: P [barcode P03206032]). [Note E]

#### Distribution.

Vietnam (Nha Trang).

#### Note E.

Vidal (1968) described *Rhaphiolepisbrevipetiolata* and cited the collection *E. Poilane 3464* deposited in herbarium P as type in the protologue, however, we found two duplicates in the herbarium P. A lectotype is needed to be chosen from these two duplicates (Turland et al. 2018: Art. 9.15). We herein designated the sheet (P03206033) that was annotated by Vidal as the lectotype. Furthermore, this species has never been well treated since its publication. Although we herein recognized this species, a further study will be needed to clarify the identity of *R.brevipetiolata*.

### 6. Rhaphiolepis cavaleriei

Taxon classificationPlantaeRosalesRosaceae

(H.Lév.) B.B.Liu & J.Wen, Front. Plant Sci. 10-1731: 10. 2020.

085077E4-12F9-536F-9346-BACDF6CD2E9B

≡ Hiptagecavaleriei H.Lév., Repert. Spec. Nov. Regni Veg. 10: 372. 15 March 1912. Type: China. Kouy-Tcheou (Guizhou): Pin-fa, montagne en pente, 20 May 1907, *J. Cavalerie 3220* (lectotype, designated by Liu et al. 2020c, pg. 112: E [barcode E00011330]!; isolectotypes: A [barcode 00055347]!, E [barcode E00284669]!, K [barcode K000758387]!, P [barcode P02143258, P02143259]!).
≡ Eriobotryacavaleriei (H.Lév.) Rehder, J. Arnold Arbor. 13: 307. 1932. Type: Based on Hiptagecavaleriei.
≡ Pyrusathenae M.F.Fay & Christenh., Global Fl. 4: 96. 2018. Type: Based on Hiptagecavaleriei.
= Eriobotryagrandiflora Rehder & E.H.Wilson, Pl. Wilson. (Sargent) 1(2): 193. 30 April 1912. Type: China. Western Szech’uan (Sichuan): alt. 1600m, May 1904, *E.H. Wilson 3506* (lectotype, designated by Liu et al. 2020c, pg. 107: A [barcode 00026472]!; isolectotypes: A [barcode 00026473]!, BM [barcode BM000602187]!, HBG [barcode HBG511040]!, K [barcode K000758386]!, P [barcode P02143267]!).
≡ Eriobotryadeflexa(Hemsl.)Nakaivar.grandiflora (Rehder & E.H.Wilson) Nakai, J. Arnold Arbor. 5(2): 72. 1924. Type: Based on Eriobotryagrandiflora.
= Eriobotryabrackloi Hand.-Mazz., Anz. Akad. Wiss. Wien, Math.-Naturwiss. Kl. 59: 102. 1922. Type: China. Kwangtung (Guangdong): In silva ad austro-occid. jugi Tsatmukngao prope oppidum Lienping ad bor.-or. urbis Kanton sita ad rivos, 800 m, substr. crystallino, 15, 27 July 1920, *R.E. Mell 659* (lectotype, designated by Liu et al. 2020c, pg. 104: WU [barcode WU0059394]!; isolectotype: A [barcode 00026469]!).
≡ Eriobotryacavaleriei(H.Lév.)Rehdervar.brackloi (Hand.-Mazz.) Rehder, J. Arnold Arbor. 13(3): 308. 1932. Type: Based on Eriobotryabrackloi.
= EriobotryabrackloiHand.-Mazz.var.atrichophylla Hand.-Mazz., Anz. Akad. Wiss. Wien, Math.-Naturwiss. Kl. 59: 103. 1922. Type: China. Hunan: austro-occ.: In monte Yün-scha prope urbem Wukang, in silva elata frondosa umbrosa. alt. 950 m, 6 June 1918, *H.F. von Handel-Mazzetti 12032* (lectotype, designated by Liu et al. 2020c, pg. 104: WU [barcode WU0059395]!; isolectotype: A [barcode 00026471]!).

#### Distribution.

China (Fujian, Guangdong, Guangxi, Guizhou, Hubei, Hunan, Jiangxi, and Sichuan) and North Vietnam (Hòa Bình and Lao Cai).

### 7. Rhaphiolepis condaoensis

Taxon classificationPlantaeRosalesRosaceae

(X.F.Gao, Idrees & T.V.Do) B.B.Liu & J.Wen, Front. Plant Sci. 10-1731: 10. 2020.

4885A025-8049-5C9E-9AC0-F66D7B4F5DD9

≡ Eriobotryacondaoensis X.F.Gao, Idrees & T.V.Do, Phytotaxa 365(3): 290. 2018. Type: Vietnam. Ba Ria-Vung Tau Province: Con Dao National Park, growing on the slope of hill under tropical evergreen forest, 20m, 8°41'30"N, 106°38'00"E, 21 March 2017, *T.V.Do VNMN_CN 633* (holotype: VNMN!; isotype: CDBI!).

#### Distribution.

Southeast Vietnam (Ba Ria-Vung Tau: Con Dao National Park).

### 8. Rhaphiolepis × daduheensis

Taxon classificationPlantaeRosalesRosaceae

(H.Z.Zhang ex W.B.Liao, Q.Fan & M.Y.Ding) B.B.Liu & J.Wen, Front. Plant Sci. 10-1731: 10. 2020.

5938F837-4EE4-5425-9132-97C6B5ACCA3F

≡ Eriobotrya×daduheensis H.Z.Zhang ex W.B.Liao, Q.Fan & M.Y.Ding, Phytotaxa 212(1): 97. 2015. Type. China. Sichuan: Hanyuan County, Dashu Town, Xinmin Village, Mt. Shizishan, in the forest edge at the foot of the mountain, 970 m, 29°17'48.18"N, 102°39'44.94"E, 19 December 2007, *Q. Fan 9292* (holotype: SYS [barcode 190936]!; isotypes: SYS!, IBSC!).

#### Distribution.

As a putative natural hybrid between *Rhaphiolepisbibas* (=*Eriobotryajaponica*) and *R.prinoides* (=*E.prinoides*), this species is restricted to Daduhe River Basin in Sichuan, China (Ding et al. 2015).

### 9. Rhaphiolepis deflexa

Taxon classificationPlantaeRosalesRosaceae

(Hemsl.) B.B.Liu & J.Wen, Front. Plant Sci. 10-1731: 10. 2020.

EF0CF6C3-FE36-52A9-A4CD-941747247357

≡ Photiniadeflexa Hemsl., Ann. Bot. 9: 153. 1895. Type: China. Formosa (Taiwan): Bankinsing, May 1894, *A. Henry 498* (lectotype, designated by Vidal 1965, pg. 566: K [barcode K000758389]! “type”; isolectotype: A [barcode 00026740]!, “isotype”).
≡ Eriobotryadeflexa (Hemsl.) Nakai, Bot. Mag. (Tokyo) 30: 18, in adnot. 1916. Type: Based on Photiniadeflexa.
= Photiniabuisanensis Hayata, Icon. Pl. Formosan. 3: 100. 1913. Type: not designated.
≡ Eriobotryadeflexa(Hemsl.)Nakaif.buisanensis (Hayata) Nakai, Bot. Mag. (Tokyo) 30(349): 18. 1916. Type: Based on Photiniabuisanensis.
≡ Eriobotryabuisanensis (Hayata) Kaneh., Formosan Trees 218. 1918. Type: Based on Photiniabuisanensis.
≡ EriobotryadeflexaNakaivar.buisanensis (Hayata) Hayata, Catal. Governm. Herb. Formos. 246. 1930. Type: Based on Photiniabuisanensis.
≡ Eriobotryabuisanensis (Hayata) Makino & Nemoto, Fl. Japan., ed. 2 (Makino & Nemoto) 464. 1931. Type: Based on Photiniabuisanensis.
= EriobotryadeflexaNakaivar.koshunensis Kaneh. & Sasaki, Catal. Gov’t Herb. Formosa 246. 1930. Type: not designated.
≡ EriobotryadeflexaNakaif.koshunensis (Kaneh. & Sasaki) H.L.Li, Lloydia 14(4): 232. 1951. Type: Based on Eriobotryadeflexavar.koshunensis.

#### Distribution.

China (Guangdong, Hainan, and Taiwan) and Vietnam (Nha Trang).

### 10. Rhaphiolepis dubia

Taxon classificationPlantaeRosalesRosaceae

(Lindl.) B.B.Liu & J.Wen, Front. Plant Sci. 10-1731: 10. 2020.

F1B350E5-4506-5EA4-8874-593B14487659

≡ Photiniadubia Lindl., Trans. Linn. Soc. London 13(1): 104, t. 10. 1821. Type: Nepal. *N. Wallich 668.1* (neotype, designated by Liu et al. 2020c, pg. 113: K [barcode K001111549]!; isoneotypes: BM [barcode BM000521995]!, E [barcode E00011335]!).
≡ Eriobotryadubia (Lindl.) Decne., in Nouv. Arch. Mus. Hist. Nat. Ser. I, x. 145. 1874. Type: Based on Photiniadubia.
= Mespilustinctoria D.Don, Prodr. Fl. Nepal. 238. 1825. Type: not designated.

#### Distribution.

Bhutan, India (Sikkim), Myanmar (Kachin, Mandalay, and Shan), and Nepal.

### 11. Rhaphiolepis elliptica

Taxon classificationPlantaeRosalesRosaceae

(Lindl.) B.B.Liu & J.Wen, Front. Plant Sci. 10-1731: 10. 2020.

1AD90E52-5014-588F-89F0-4BEF049D99E9

≡ Eriobotryaelliptica Lindl., Trans. Linn. Soc. London 13(1): 102. 1821. Type: Nepal. Narainhetty. 1 February 1803, *F. Buchanan-Hamilton s.n.* (holotype: BM [barcode BM000521994]!).
≡ Cotoneasterellipticus (Lindl.) Loudon, Encyc. Pl. 1208. 1841. Type: Based on Eriobotryaelliptica.
≡ Pyruselliptica (Lindl.) M.F.Fay & Christenh., Global Fl. 4: 102. 2018. Type: Based on Eriobotryaelliptica.

### 11a. Rhaphiolepis elliptica var. elliptica

Taxon classificationPlantaeRosalesRosaceae

AD3F8396-0802-5F55-8B18-1836CA522D9D

= Mespiluscuila Buch.-Ham. ex D.Don, Prodr. Fl. Nepal. 238. 1825, nom. nov. superfl. Type: Based on Eriobotryaelliptica Lindl.

#### Distribution.

China (Tibet) and Nepal (Narainhetty).

### 11b. Rhaphiolepis elliptica (Lindl.) B.B.Liu & J.Wen var. petelotii

Taxon classificationPlantaeRosalesRosaceae

(J.E.Vidal) B.B.Liu & J.Wen, Front. Plant Sci. 10-1731: 10. 2020.

568F6D33-643E-535E-A336-DFF44B68F0F9

≡ EriobotryaellipticaLindl.var.petelotii J.E.Vidal, Adansonia sér. 2, 5: 552. 1965. Type: Vietnam. “prov. de Lao Kay, Chapa, 1500 m”, January 1929, *M. Pételot s.n.* (lectotype, designated by Liu et al. 2020c, pg. 105: P [barcode P02143261]!; isolectotype: P [barcode P02143262]!).

#### Distribution.

N Vietnam (Lao Cai).

### 12. Rhaphiolepis ferruginea

Taxon classificationPlantaeRosalesRosaceae

F.P.Metcalf, Lingnan Sci. J. 18: 509. 1939, “ Raphiolepis”.

5B871DAB-6B0D-5E9A-BA5B-AD11B448ED98

≡ Pyrussodomacea M.F.Fay & Christenh., Global Fl. 4: 121. 2018. Type: China. Guangdong: Tapu District, Tung Koo Shan, 8–29 September 1932, *W.T. Tsang 21587* (holotype: A [barcode 00032516]!; isotypes: K [barcode K000758194]!, P [barcode P02143130]!).

### 12a. Rhaphiolepis ferruginea var. ferruginea

Taxon classificationPlantaeRosalesRosaceae

19D092BC-951E-5030-B255-DBE8D86CB570

#### Distribution.

China (Fujian, Guangdong, Guangxi, and Hainan).

### 12b. Rhaphiolepis ferruginea F.P.Metcalf var. serrata

Taxon classificationPlantaeRosalesRosaceae

F.P.Metcalf, Lingnan Sci. J. 18: 511. 1939, “ Raphiolepis”.

8AD6499D-9607-51ED-AC5A-2FF6B7A80A71

#### Type.

China. Guangdong: Lung-tau Mt., near Iu Village, May 22-July 5, 1924, *To & Tsang 12546* (**lectotype, designated here**: A [barcode 00032518]!; isolectotype: A [barcode 00032517, 00032519]!). [Note F]

#### Distribution.

China (Fujian, Guangdong, and Guangxi).

#### Note F.

*To & Tsang 12546* was designated as the type in the protologue. We located three specimens in herbarium A, therefore, they are syntypes. A subsequent lectotypification is necessary. We designated the sheet (A00032518) in a better condition as the lectotype herein.

### 13. Rhaphiolepis fulvicoma

Taxon classificationPlantaeRosalesRosaceae

(Chun ex W.B.Liao, F.F.Li & D.F.Cui) B.B.Liu & J.Wen, Front. Plant Sci. 10-1731: 10. 2020.

8280A667-65B5-5D80-8A43-6EE53E9CAA2F

≡ Eriobotryafulvicoma Chun ex W.B.Liao, F.F.Li & D.F.Cui, Ann. Bot. Fenn. 49(4): 264. 2012. Type: China. Guangdong: Xinyi County, Dawuling Natural Reserve, 45 m, 28 April 1932, *Z. Huang 32257* (holotype: WUK [barcode 0109531]!; isotypes: IBK [barcode IBK00060958, IBK00060976]!, IBSC [barcode 0298975]!, KUN [barcode 0116268]!, PE [barcode 00799336]!, SZ [barcode 00194329]!).

#### Distribution.

China (Guangdong).

### 14. Rhaphiolepis glabrescens

Taxon classificationPlantaeRosalesRosaceae

(J.E.Vidal) B.B.Liu & J.Wen, Front. Plant Sci. 10-1731: 11. 2020.

A52408EB-EB3D-545A-803A-AFA17FD8B272

≡ Eriobotryaglabrescens J.E.Vidal, Adansonia sér. 2, 5: 554. 1965. Type: Myanmar. Kachin State: “N. Birmanie, Triangle, Hkinlum village, 2500 m, en fleurs”, 4 April 1953, *F. Kingdon-Ward 20616* (lectotype, designated by Liu et al. 2020c, pg. 106: BM [barcode BM000602189]; isolectotypes: A [barcode 00026482]!, E [barcode E00011336]!).
≡ Pyrusserpentae M.F.Fay & Christenh., Global Fl. 4: 121. 2018. Type: Based on Eriobotryaglabrescens.

### 14a. Rhaphiolepis glabrescens var. glabrescens

Taxon classificationPlantaeRosalesRosaceae

401BFF79-D4F6-5D5D-8697-562E936AA391

#### Distribution.

North Myanmar (Triangle, Centre Ouest, and Khai Yang).

### 14b. Rhaphiolepis glabrescens (J.E.Vidal) B.B.Liu & J.Wen var. victoriensis

Taxon classificationPlantaeRosalesRosaceae

(J.E.Vidal) B.B.Liu & J.Wen, Front. Plant Sci. 10-1731: 11. 2020.

B3692F3C-749A-59A4-A755-E5499A7ACD63

≡ EriobotryaglabrescensJ.E.Vidalvar.victoriensis J.E.Vidal, Adansonia sér. 2, 5: 555. 1965. Type: Myanmar. KachinState: “Birmanie centrale, Mt Victoria, 3000 m, en fleurs”, 2 April 1956, *F. Kingdon-Ward 21915* (holotype: BM [barcode BM000602190]!).

#### Distribution.

North Myanmar (Centre Ouest: Mt Victoria).

### 15. Rhaphiolepis henryi

Taxon classificationPlantaeRosalesRosaceae

(Nakai) B.B.Liu & J.Wen, Front. Plant Sci. 10-1731: 11. 2020.

B0FEC08D-2F6E-554B-AB36-65E046B87518

≡ Eriobotryahenryi Nakai, J. Arnold Arbor. 5: 70. 1924. Type: China. Yunnan: Szemao (Simao), 1900, *A. Henry 13018* (lectotype, selected by Vidal 1965, pg. 562, first step “type”; second step, designated by Liu et al. 2020c, pg. 107: A [barcode 00026474]!; isolectotypes: K [barcode K000758388]!, NY [barcode 00436209]!).
≡ Pyrushenryi (Nakai) M.F.Fay & Christenh., Global Fl. 4:106. 2018. Type: Based on Eriobotryahenryi.

#### Distribution.

China (Guizhou and Yunnan) and Myanmar (Pyin Oo Lwin).

### 16. Rhaphiolepis hookeriana

Taxon classificationPlantaeRosalesRosaceae

(Decne.) B.B.Liu & J.Wen, Front. Plant Sci. 10-1731: 11. 2020.

C7F4CC95-BB8D-509B-A275-B3E096A9E5C0

≡ Eriobotryahookeriana Decne., Nouv. Arch. Mus. Hist. Nat. Ser. I 10:146. 1874. Type: India. Sikkim: Jongri, 13000–15000 ft., 10 August 1862, *T. Anderson 490* (lectotype, designated by Vidal 1965, pg. 563: P [barcode P02143268]!, “type”; isolectotype: GH [barcode 00026483]!, “isotype”).
≡ Pyrushookeriana (Decne.) M.F.Fay & Christenh., Global Fl. 4: 107. 2018. Type: Based on Eriobotryahookeriana.

#### Distribution.

Bhutan and India (Sikkim).

### 17. Rhaphiolepis indica

Taxon classificationPlantaeRosalesRosaceae

(L.) Lindl., Bot. Reg. 6: t. 468. 1820.

66FCF527-C989-503D-9484-59BDEF50114E

≡ Crataegusindica L., Sp. Pl. 1: 477. 1753. Type: India. *s.coll. s.n.* (lectotype, designated by Vidal 1968, pg. 85: LINN [barcode LINN-HL643-11]! “type”). [Note G]

#### Note G.

Vidal (1968) provided the lectotype for *Crataegusindica*, while he wrote it as type. Jarvis (2007) confirmed this typification and corrected it as lectotype.

### 17a. Rhaphiolepis indica var. indica

Taxon classificationPlantaeRosalesRosaceae

30910F3E-7609-5F37-9D92-62C34CD8E7F2

= Crataegusrubra Lour., Fl. Cochinch. 1: 320. 1790. Type: China. Guangdong: “Habitat agrestis prope Cantone Sinarum”, *J. Loureiro 320-3* (holotype: P [barcode P00150873]!).
≡ Mespilusrubra (Lour.) Stokes, Bot. Mat. Med. iii. 110. 1812. Type: Based on Crataegusrubra.
≡ Rhaphiolepisrubra (Lour.) Lindl., Coll. Bot. (Lindley) t. 3. 1821. Type: Based on Crataegusrubra.
= Mespilussinensis Poir., Encyc. [J. Lamarck & al.] Suppl. 4. 70. 1816. Type: not designated.
≡ Crataegussinensis (Poir.) Loisel., Herb. Amat. iv. t. 247. 1820. Type: Based on Mespilussinensis.
≡ Rhaphiolepissinensis (Poir.) M.Roem., Syn. Rosifl. 3: 114. 1847. Based on Mespilussinensis.
= Opametrosideros Lour., Fl. Cochinch. 1: 309. 1790. Type: “Cochinchina”, *J. Loureiro s.n.* (holotype: BM [barcode BM000906022]!).
≡ Syzygiummetrosideros (Lour.) DC., Prodr. [A. P. de Candolle] 3: 261. 1828. Type: Based on Opametrosideros.
≡ Eriobotryametrosideros (Lour.) A.Chev., Cat. Pl. Jard. Bot. Saigon 64. 1919. Type: Based on Opametrosideros.
= Rhaphiolepiscrataegoides M.Roem., Syn. Rosifl. 113. 1847. Type: not designated.
≡ Rhaphiolepisindica(L.)Lindl.var.crataegoides (M.Roem.) Nakai, J. Arnold Arbor. 5: 66. 1924. syn. nov. Type: Based on Rhaphiolepiscrataegoides.
= Rhaphiolepisfragrans E.T.Geddes, Bull. Misc. Inform. Kew 1929(4): 108. 1929, “Raphiolepis”. Type: Thailand. Kemarat, Ubon, Kan Kak, 100 m, 30 January 1924, *A.F.G. Kerr 8257A* (**lectotype, designated here**: K [barcode K000758246]!; isolectotypes: BM [barcode BM000602125]!, C [barcode C10017919]!, E [barcode E00011337]!). Siam: 100 m, January 1924, *A.F.G. Kerr 8257* (paratype: TCD [barcode TCD0016617]!) [Note H]
= Rhaphiolepisgracilis Nakai, J. Arnold Arbor. 5: 64. 1924. Type: China. Zhejiang: S. Yentang, 600 ft (ca. 183 m), August 26, 1920, *H.H. Hu 228* (**lectotype, designated here**: A [barcode 00032521]!). ibidem, 10 ft, August 24, 1920, *H.H. Hu 220* (syntype: A [barcode 00032520]!). [Note I]
= Rhaphiolepisindica(L.)Lindl.var.latifolia Cardot, Notul. Syst. (Paris) 3: 380. 1918. Type: not designated.
= Rhaphiolepisindica(L.)Lindl.var.mekongensis Cardot, Notul. Syst. (Paris) 3: 380. 1918. Type: Laos. “Bassin du Mékong: rivière Selamphao”, 1876, *Harmand 202* (holotype: A [barcode 00032551]!).
≡ Rhaphiolepismekongensis (Cardot) Tagane & H.Toyama, Acta Phytotax. Geobot. 66(2): 127. 2015. Type: Based on Rhaphiolepisindicavar.mekongensis.
≡ Pyrusmekongensis (Cardot) M.F.Fay & Christenh., Global Fl. 4: 112. 2018. Type: Based on Rhaphiolepisindicavar.mekongensis.
= Rhaphiolepiskerrii E.T.Geddes, Bull. Misc. Inform. Kew 4: 109. 1929, “Raphiolepis”. Type: Thailand. Siam: “Kao Krading, 1200 m”, 12 March 1924, *A.F.G. Kerr 8689* (**lectotype, designated here**: K [barcode K000758247]!; isolectotypes: BK (barcode BK257293)!, BM (barcode BM000602126)!). [Note J]
= Rhaphiolepisloureiroi Spreng., Syst. Veg., ed. 16 [Sprengel] 2: 508. 1825. Type: not designated.
= Rhaphiolepisparvibracteolata Merr., Philipp. J. Sci. 21: 344. 1922. Type: China. Hainan: Nodoa, roadside in wilderness, 250 m, 2 January 1922, *F.A. McClure 8015* (**lectotype, designated here**: US [barcode 00097487]!; isolectotypes: A [barcode 00032548, 00032549]!, NY [barcode 00415903]!). [Note K]
= Rhaphiolepisrubra(Lour.)Lindl.var.foliosa Nakai, J. Arnold Arbor. 5: 66. 1924. syn. nov. Type: not designated.
= Rhaphiolepisrubra(Lour.)Lindl.var.lanceolata Nakai, J. Arnold Arbor. 5: 67. 1924. syn. nov. Type: not designated.
= Rhaphiolepisrugosa Nakai, J. Arnold Arbor. 5: 62. 1924. Type: China. Jiangxi: Anfu County, Woo Kung Shan, 3500 ft, 20 April 1921, *H.H. Hu 711* (holotype: A [barcode 00032550]!).

#### Distribution.

Cambodia, China, Indonesia, Japan, Laos, Thailand, and Vietnam.

#### Note H.

Geddes (1929) described *Rhaphiolepisfragrans* and designated “*Kerr 8257A*” as the type. However, we located four sheets in four different herbaria (BM, C, E, K), all of which represent duplicates from a homogeneous collection. A lectotype is needed to be chosen from these four duplicates (Turland et al. 2018: Art. 9.15). We designated the duplicate in K (K000758246) in a better condition as the lectotype herein. It should be noted that Geddes (1930) described another name, *Pyrusfragrans* E.T.Geddes (in Bull. Misc. Inform. Kew 4: 161. 1930) with the same epithet.

#### Note I.

Nakai (1924) described *Rhaphiolepisgracilis* and designated two collections as type, therefore, they are syntypes and a lectotype is necessary to be chosen from them (Art. 9.12: Turland et al. 2018). We designated the collection, *H.H. Hu 228* (A00032521) as the lectotype, as it is deposited in a better condition.

#### Note J.

Geddes (1929) designated “*Kerr 8689*” as the type in his work “Contributions to the flora of Siam. Additamentum XXVI”. We found three sheets of this collection in herbaria BK, BM, and K. According to Art. 9.15 (Turland et al. 2018), it is necessary for us to choose one of these three specimens as lectotype. We lectotypified the duplicate in K (K000758247) for *Rhaphiolepiskerrii*, as it was preserved in a better condition.

#### Note K.

Merrill (1922) described *Rhaphiolepisparvibracteolata* and designated the collection, *F.A. McClure 8015*, as the type. However, we located four sheets in herbaria A, NY, and US, from which the lectotype could be chosen. As the duplicate deposited in the herbarium US (00097487) has the identification tag of “......Rhaphiolepisparvibracteolata Merr. n. sp. ...... IDENTIFIED BY E. D. MERRILL ......”, we herein designated this sheet as the lectotype.

### 17b. Rhaphiolepis indica (L.) Lindl. var. phaeostemon

Taxon classificationPlantaeRosalesRosaceae

(Lindl.) Nakai, J. Arnold Arbor. 5: 65–66. 1924.

0AD9CB3C-DD83-5BB9-97C8-FA510DB3FF62

≡ Rhaphiolepisphaeostemon Lindl., Coll. Bot. (Lindley) sub t. 3. 1821. Type. Not designated.

#### Distribution.

China.

### 17c. Rhaphiolepis indica (L.) Lindl. var. shilanensis

Taxon classificationPlantaeRosalesRosaceae

Yuen P.Yang & H.Y.Liu, Taiwania 47(2): 176. 2002.

532F27DE-B5F8-5E40-9000-6C00C6123354

#### Type.

China. Taiwan: Pingtung County, Nanjenshan, October 25, 1978, *K.S. Hsu & Y.P. Yang s.n.* (holotype: TAIF).

#### Distribution.

China (Taiwan).

### 17d. Rhaphiolepis indica (L.) Lindl. var. spiralis

Taxon classificationPlantaeRosalesRosaceae

(Blume) Nakai, J. Arnold Arbor. 4: 65. 1924.

6080ACCC-CCE3-5ACC-9339-CA39E826326A

≡ Mespilusspiralis Blume, Bijdr. Fl. Ned. Ind. 17: 1102, 1826. Type: Indonesia. Java: *C.L. Blume s.n.* (**lectotype, designated here**: L [barcode L0019710]!; isolectotypes: L [barcode L0019711, L0019712, L0019713]!, NY [barcode 00436082]!). [Note L]
≡ Rhaphiolepisspiralis (Blume) G.Don, Gen. Hist. 2: 602. 1832. Type: Based on Mespilusspiralis.
≡ Crataegusspiralis (Blume) Steud., Nomencl. Bot. [Steudel], ed. 2. i. 434. 1841. Type: Based on Mespilusspiralis.
≡ Opaspiralis (Blume) Seem., J. Bot. 1: 281. 1863. Type: Based on Mespilusspiralis.

#### Distribution.

Indonesia (Java).

#### Note L.

Blume (1826) described *Mespilusspiralis* in his book “Bijdragen tot de flora van Nederlandsch Indië”, noting that the original material on which it was based was collected from a plant introduced from China in Java. We found five sheets representing the duplicates from one collection, four of them from herbarium L and one of them from herbarium NY. It will be necessary for us to choose one of them as the lectotype. According to Stafleu and Cowan (1976), Blume’s original collections were deposited at L, second set at BO, and type mainly at L, but also at BO and P. One of the four duplicates deposited at L, therefore, will be a candidate for the lectotype. We designated the sheet (L0019710) in a better condition as the lectotype.

### 17e. Rhaphiolepis indica (L.) Lindl. var. tashiroi

Taxon classificationPlantaeRosalesRosaceae

Hayata ex Matsum. & Hayata, J. Coll. Sci. Imp. Univ. Tokyo 22: 129. 1906.

19EEC945-646D-5646-9D2B-D3C88B94D358

#### Type.

Not designated.

#### Distribution.

China (Taiwan).

### 17f. Rhaphiolepis indica (L.) Lindl. f. impressivena

Taxon classificationPlantaeRosalesRosaceae

(Masam.) S.S.Ying, Coloured Illustr. Fl. Taiwan 1: 371. 1985.

25CCDD6D-F808-56F5-AAF2-32AFD6B1CFA8

≡ Rhaphiolepisimpressivena Masam., Trans. Nat. Hist. Soc. Formosa 30: 340. 1940. Type: China. Taiwan: “In rocky place between 1000–1200 m, Mt. Seisui-zan (Chingshui), Karengun, April 8, 1939”, *T. Nakamura 365* (holotype: TI).

#### Distribution.

China (Taiwan).

### 17g. Rhaphiolepis indica (L.) Lindl. f. minor

Taxon classificationPlantaeRosalesRosaceae

(Makino) H.Ohashi, J. Jap. Bot. 63(1): 6. 1988.

236C8438-293D-5C6D-94BB-49CDC325EB83

≡ Rhaphiolepisumbellata(Thunb.)Makinovar.minor Makino, Bot. Mag. (Tokyo) 16: 14. 1902. Type: Japan. Prov. Mushashi: Tokyo, Bot. Gard. Koishikawa, cult. 19 May 1880, *T. Makino s.n.* (syntype: TI); 20 May 1890, T. Makino s.n. (syntype: TI); May 1896, *T. Makino s.n.* (syntype: TI).
≡ Rhaphiolepisminor (Makino) Koidz., Bot. Mag. (Tokyo) 23: 171. 1909. Type: Based on Rhaphiolepisumbellatavar.minor.
≡ Rhaphiolepisrubra(Lour.)Lindl.var.minor (Makino) Nakai, J. Arnold Arbor. 5: 67. 1924. Type: Based on Rhaphiolepisumbellatavar.minor.
≡ Rhaphiolepisindica(L.)Lindl.var.minor (Makino) Kitam., Acta Phytotax. Geobot. 26(1–2): 2. 1974. Type: Based on Rhaphiolepisumbellatavar.minor.

#### Distribution.

Japan (Tokyo).

### 18. Rhaphiolepis integerrima

Taxon classificationPlantaeRosalesRosaceae

Hook. & Arn., Bot. Beechey Voy. 263. 1838.

743BEE5E-44BD-513E-BD54-F0FDD7229A4B

≡ Opaintegerrima (Hook. & Arn.) Seem., J. Bot. 1: 281. 1863. Type: Japan. “Bonin [in] Loa Choo”, *Beechey s.n.* (**lectotype, designated here**: K [barcode K000758198]!; isolectotypes: K [barcode K000758197, K000758199, K000758200]!).
≡ RhaphiolepisjaponicaSiebold & Zucc.var.integerrima (Hook. & Arn.) Hook.f., Bot. Mag. 91: pl. 5510. 1865. Type: Based on Opaintegerrima.
≡ Rhaphiolepisintegerrima (Hook. & Arn.) Hort., ex Handl. Trees Kew Pt. i. [Polypet.] 217. 1894. Type: Based on Opaintegerrima.
≡ Rhaphiolepisumbellata(Thunb.)Makinof.integerrima Rehder, Mitt. Deutsch. Dendrol. Ges. 24: 223. 1915. Type: Based on Opaintegerrima.
≡ Rhaphiolepisumbellata(Thunb.)Makinovar.integerrima (Hook. & Arn.) Masam., Sci. Rep. Kanazawa Univ. 3: 3. 1955. Type: Based on Opaintegerrima.
≡ Pyrusgodiva M.F.Fay & Christenh., Global Fl. 4: 105. 2018. Type: Based on Opaintegerrima.
= Rhaphiolepismertensii Siebold & Zucc., Fl. Jap. (Siebold) 1: 164. 1841. Type: not designated.
≡Opamertensii (Siebold & Zucc.) Seem., J. Bot. 1: 281. 1863. Type: Based on Rhaphiolepismertensii.
≡ Rhaphiolepisumbellata(Thunb.)Makinovar.mertensii (Siebold & Zucc.) Makino, Bot. Mag. (Tokyo), 16(179): 14. 1902. Type: Based on Rhaphiolepismertensii.
≡ RhaphiolepisintegerrimaHook. & Arn.var.mertensii (Siebold & Zucc.) Makino ex Koidz., J. Coll. Sci. Imp. Univ. Tokyo 34(2): 72. 1913. Type: Based on Rhaphiolepismertensii.

#### Distribution.

China (Taiwan) and Japan (Ryukyu Islands).

### 19. Rhaphiolepis jiulongjiangensis

Taxon classificationPlantaeRosalesRosaceae

P.C.Huang & K.M.Li, J. Nanjing Forest. Univ. 13(4): 85. fig. IA, 1989, as ‘ Raphiolepis’.

79D2877A-222E-5DBC-A497-1C488B8B7657

≡ Pyrusjiulongjiangensis (P.C.Huang & K.M.Li) M.F.Fay & Christenh., Global Fl. :4: 108. 2018. Type. China. Fujian: Hua’an County, Jiulongjiang, 120 m, April 1987, *K.M. Li 30439* (holotype: NJF).

#### Distribution.

China (Fujian).

### 20. Rhaphiolepis lanceolata

Taxon classificationPlantaeRosalesRosaceae

Hu, J. Arnold Arbor. 13: 335. 1932.

4162B183-E84A-5D72-96FB-C3930DA0478C

≡ Pyruslanceolata (Hu) M.F.Fay & Christenh., Global Fl. 4: 110. 2018. Type: China. Guangxi: “Seh-feng Dar-shan, S. Nanning, alt. 775 m”, 21 Oct. 1928, *R.C. Ching 8060* (holotype: A [barcode 00032543]!; isotypes: A [barcode 00032544, 00032545]!, NY [barcode 00415902]!).
= Rhaphiolepishainanensis F.P.Metcalf, Lingnan Sci. J. 18: 511. 1939. “Raphiolepis” Type: China. Hainan: Po-ting, 1300 ft, September 25, 1935, *F.C. How 73712* (**lectotype, designated here**: A [barcode 00032523]!). Dung Ka to Wen Fa Shi, 1700 ft, March 3, 1932, *N.K. Chun & C.L. Tso 43670* (syntypes: A [barcode 00032522]!, P [barcode P02143129]!). Yaichow, July 9, 1933, *H.Y. Liang 61989* (paratype). [Note M]
= Rhaphiolepisindica(L.)Lindl.var.angustifolia Cardot, Notul. Syst. (Paris) 3: 380. 1918. Type: Vietnam. “province de Quant-tri, vallée de haute rivière de Cu-Bi”, *Eberhardt 2057* (holotype: P; isotypes: A [barcode 00032540]!).

#### Distribution.

China (Guangdong?, Guangxi, and Hainan).

#### Note M.

In the protologue of *Rhaphiolepishainanensis*, two specimens was designated as types, they are *N.K. Chun & C.L. Tso 43670* as “type flower”, and *F.C. How 73712* as “type fruit”, which are therefore syntypes. Metcalf also cited the specimens *H.Y. Liang 61989* but without designating it as a type; it is, therefore, a paratype. It is necessary to choose one specimen as the lectotype from the two types; we, therefore, designated the “type fruit” specimen “*F.C. How 73712*” in a better condition as the lectotype herein.

### 21. Rhaphiolepis laoshanica

Taxon classificationPlantaeRosalesRosaceae

(W.B.Liao, Q.Fan & S.F.Chen) B.B.Liu & J.Wen comb. nov.

3BF8DC2F-9021-5E1A-8569-E0EABBB4A8BA

urn:lsid:ipni.org:names:77210700-1

≡ Eriobotryalaoshanica W.B.Liao, Q.Fan & S.F.Chen, PhytoKeys 146: 64 (2020). Type: China. Yunnan: Malipo County, Mount Laoshan, in thin forests on the slopes of limestone hills, 22°59.08'N, 104°50.48'E, 1160 m a.s.l., 14 October 2019, *Q. Fan 17570* (holotype: SYS; isotypes: IBSC, SYS).

#### Distribution.

China (Yunnan).

### 22. Rhaphiolepis latifolia

Taxon classificationPlantaeRosalesRosaceae

(Hook.f.) B.B.Liu & J.Wen, comb. nov. [Note N]

A7478976-4078-5A61-B9BE-01741615CDEE

urn:lsid:ipni.org:names:77210701-1

≡ Eriobotryalatifolia Hook.f., Fl. Brit. India [J. D. Hooker] 2(5): 370. 1878. Type: Myanmar. Moalmayne, on Thoung Gyne, alt. 5000 ft., 1857, *T. Lobb s.n.* (holotype: K [barcode K000758400]!).
≡ Pyrusherae M.F.Fay & Christenh., Global Fl. 4: 106. 2018. Type: Based on Eriobotryalatifolia.
≡ Rhaphiolepisherae (M.F.Fay & Christenh.) B.B.Liu & J.Wen, Front. Plant Sci. 10-1731: 11. 2020. Type: Based on Eriobotryalatifolia.

#### Distribution.

Myanmar (Kayin and Taninthayi).

#### Note N.

*Rhaphiolepislatifolia* Lodd. ex G.Don (in Hort. Brit. [Loudon] 202. 1830) was a naked name because this name has been published without descriptive statements in the protologue (Turland et al. 2018: Art. 38.1). *Rhaphiolepislatifolia* has thus not been occupied. Although Liu et al. (2020b) made a new combination for this name as *R.herae* (M.F.Fay & Christenh.) B.B.Liu & J.Wen, the correct name of this taxon should be *R.latifolia*. We need to make another new combination as *Rhaphiolepislatifolia* (Hook.f.) B.B.Liu & J.Wen herein.

### 23. Rhaphiolepis longifolia

Taxon classificationPlantaeRosalesRosaceae

(Decne.) B.B.Liu & J.Wen, Front. Plant Sci. 10-1731: 11. 2020.

C094BDFE-C538-5A19-BCDA-F1B6D35B6188

≡ Photinialongifolia Decne., Nouv. Arch. Mus. Hist. Nat. Ser. I 10: 142. 1874. Type: Bangladesh. East Bengal. Mishmi Hills, *W. Griffith 2093* (lectotype, designated by Liu et al. 2020c, pg. 113: P [barcode P02143220]!; isolectotype: K [barcode K000758398]!).
≡ Eriobotryalongifolia (Decne.) Hook.f., Fl. Brit. India [J. D. Hooker] 2(5): 370. 1878. Type: Based on Photinialongifolia.

#### Distribution.

Bangladesh (East Bengal).

### 24. Rhaphiolepis macrocarpa

Taxon classificationPlantaeRosalesRosaceae

(Kurz) B.B.Liu & J.Wen, Front. Plant Sci. 10-1731: 11. 2020.

51C7768F-7B95-58E6-8163-A464877414FE

≡ Eriobotryamacrocarpa Kurz, J. Asiat. Soc. Bengal, Pt. 2, Nat. Hist. 41(4): 306. 1872. Type: not designated.

#### Distribution.

Myanmar (Bago and Mandalay).

### 25. Rhaphiolepis major

Taxon classificationPlantaeRosalesRosaceae

Cardot, Notul. Syst. (Paris) 3: 380. 1918.

69D71100-1877-5506-AD7E-9E8682714C52

≡ Pyrusmajor (Cardot) M.F.Fay & Christenh., Global Fl. 4: 111. 2018. Type: China. Fujian: Wuyishan County, Kuantun (Guadun Village), April 1898, *M. de Latouche s.n.* (holotype: P [barcode P02143132]!; isotypes: A [barcode 00032547]!, K [barcode K000758196]!, P [barcode P02143133, P02143134]!)
= Rhaphiolepisindica(L.)Lindl.var.grandifolia Franch., Bull. Soc. Bot. France 46: 207. 1899. Type: not designated.

#### Distribution.

China (Fujian, Jiangsu, Jiangxi, and Zhejiang).

### 26. Rhaphiolepis malipoensis

Taxon classificationPlantaeRosalesRosaceae

(K.C.Kuan) B.B.Liu & J.Wen, Front. Plant Sci. 10-1731: 11. 2020.

351DF3CE-304A-5092-BABA-8B753A7844FC

≡ Eriobotryamalipoensis K.C.Kuan, Acta Phytotax. Sin. 8(3): 231. 1963. Type: China. Yunnan: Malipo County, Hwang-jin-yinn, 1200 m, 21 January 1940, *C.W. Wang et al. 86318* (holotype: PE [barcode 00004573]!; isotypes: IBSC [barcode 0299391]!, KUN [barcode 0116367]!).
≡ Pyrusmalipoensis (K.C.Kuan) M.F.Fay & Christenh., Global Fl. 4: 111. 2018. Type: Based on Eriobotryamalipoensis.

#### Distribution.

China (SE Yunnan).

### 27. Rhaphiolepis merguiensis

Taxon classificationPlantaeRosalesRosaceae

(J.E.Vidal) B.B.Liu & J.Wen, Front. Plant Sci. 10-1731: 11. 2020.

37BD40E0-93F1-5405-9DC7-4A1DC6EC8717

≡ Eriobotryamerguiensis J.E.Vidal, Adansonia sér. 2, 5: 563. 1965. Type: Myanmar. “Birmanie, Mergui, Mout Myinmolekat, 1200 m, en fruits”, 17 January 1930, *R.N. Parker 3098* (holotype: K [barcode K000758399]!).
≡ Pyrusmerguiensis (J.E.Vidal) M.F.Fay & Christenh., Global Fl. 4: 112. 2018. Type: Based on Eriobotryamerguiensis.

#### Distribution.

Myanmar (Mergui Archipelago and Taninthayi).

### 28. Rhaphiolepis oblongifolia

Taxon classificationPlantaeRosalesRosaceae

(Merr. & Rolfe) B.B.Liu & J.Wen, Front. Plant Sci. 10-1731: 11. 2020.

85E39B0C-F438-50B5-B38A-A79C72D9102D

≡ Eriobotryaoblongifolia Merr. & Rolfe, Philipp. J. Sci., C 3: 102. 1908. Type: Philippines. Mindanao. Misamis: Mount Malindang, May 1906, *E.A. Mearns & W.J. Hutchinson 4680* (lectotype, designated by Liu et al. 2020c, pg. 108: NY [barcode 00436215]!; isolectotype: US [barcode 00097490]!).

#### Distribution.

Philippines (Mindanao).

### 29. Rhaphiolepis obovata

Taxon classificationPlantaeRosalesRosaceae

(W.W.Sm.) B.B.Liu & J.Wen, Front. Plant Sci. 10-1731: 11. 2020.

CF74CF29-3F8B-54D6-B087-7C2FA10CF568

≡ Eriobotryaobovata W.W.Sm., Notes Roy. Bot. Gard. Edinburgh 10: 29. 1917. Type: China. Yunnan: in the vicinity of Yunnanfu, *E.E. Maire 2450* (holotype: E [barcode E00011331]!; isotypes: E [barcode E00284668]!, K [barcode K000758390]!).
≡ Pyrusobovata (W.W.Sm.) M.F.Fay & Christenh., Global Fl. 4: 114. 2018. Type: Based on Eriobotryaobovata.

#### Distribution.

China (C Yunnan).

### 30. Rhaphiolepis petiolata

Taxon classificationPlantaeRosalesRosaceae

(Hook.f.) B.B.Liu & J.Wen, Front. Plant Sci. 10-1731: 11. 2020.

851F9429-9013-5287-85D9-8C02B5ACAEC7

≡ Eriobotryapetiolata Hook.f., Fl. Brit. India [J. D. Hooker] 2(5): 370. 1878. Type: Sikkim, 9000 ft, *J.D. Hooker s.n.* (lectotype, designated by Liu et al. 2020c, pg. 109: K [barcode K000758394]).
≡ Pyruspetiolata (Hook.f.) M.F.Fay & Christenh., Global Fl. 4: 115. 2018. Type: Based on Eriobotryapetiolata.

#### Distribution.

Bangladesh (Chittagong), Bhutan, India (Khasia and Sikkim), and Myanmar (Chin).

### 31. Rhaphiolepis philippinensis

Taxon classificationPlantaeRosalesRosaceae

(S.Vidal) Kalkman, Blumea 21(2): 434. 1973.

9F3C80E1-DD08-51C8-88E8-0D5BE93BAB8F

≡ Eriobotryaphilippinensis S.Vidal, Revis. Pl. Vasc. Filip. 123. 1886. Type: Philippines. Luzon: “Santa Cruz, Pr. Zambales”, *S. Vidal 1350* (**lectotype, designated here**: K [barcode K000758204]!, isolectotype: MA [barcode MA729287]!). “Infanta, Pr. Zambales”, *S. Vidal 1353* (syntype: K [barcode K000758205]!, MA [barcode MA729288, MA729288-2]!). [Note O]
≡ Pyrusphilippinensis (S.Vidal) M.F.Fay & Christenh., Global Fl. 4: 115. 2018. Type: Based on Eriobotryaphilippinensis.
= Photinialuzonensis Merr., Publ. Bur. Sci. Gov. Lab. 17: 18. 1904. Type: Philippines. Luzon: Lamao River Mt. Mariveles, Provence of Bataan, Oct. 1903, *E.D. Merrill 3223* (**lectotype**, selected by Kalkman 1973, pg. 434, first step; **second step, designated here**: NY [barcode 00436125]!, isolectotypes: K [barcode K000758209]!, P [barcode P02143221]!). ibidem, January 1, 1904, *E.D. Merrill 3714* (syntypes: BM [barcode BM000602128]!, K [barcode K000758206]!). [Note P]
≡ Eriobotryaluzonensis (Merr.) Nakai, J. Arnold Arbor.5: 69. 1924. “luzoniensis”. Type: Based on Photinialuzonensis.
= Eriobotryaacuminatissima Nakai, J. Arnold Arbor. 5: 71. 1924. Type: Philippines. Luzon: Panay Province, mt. Salibongbong Capiz, June 1919, *A. Martelino & G. Edano 35622* (lectotype, designated by Liu et al. 2020c, pg. 101: A [barcode 00026487]!; isolectotypes: BM [barcode BM000602127]!, L [barcode L0019714]!, P [barcode P02143260]).

#### Distribution.

Philippines and Malaysia (Borneo: Sabah).

#### Note O.

Vidal (1886) designated two collections as the type when he described *Eriobotryaphilippinensis* in his work “Revision de Plantas Vasculares Filipinas”, therefore, they are syntypes (Art. 9.6: Turland et al. 2018). We herein select the flowering specimen (*S. Vidal 1350*) as a candidate for the lectotype, however, two duplicates of this collection were located in herbaria K and MA. We must narrow the lectotype to a single sheet. In the 1870s Vidal took up posts in the forestry service of the Philippines, however, in 1883 he was back in Europe and visited the herbaria there including K (Vidal 1885). Therefore, we herein designate the sheet of *S. Vidal 1350* in K (barcode K000758204) as the lectotype.

#### Note P.

Merrill (1904) described *Photinialuzonensis* and mentioned two collections (*E.D. Merrill 3223 & 3714*) in the protologue, and thus they are syntypes (Art. 9.6: Turland et al. 2018). Kalkman (1973) wrote as “Type: *Merrill 3223* (K, iso); paratype: *Merrill 3714* (BM, K)”, which means that he designated *Merrill 3223* as the lectotype [first-step]. We located two duplicates in herbaria NY and K, respectively, and thus it is necessary to narrow the lectotype to one single specimen. According to Stafleu and Cowan (1981), Merrill’s type and material are deposited in A, FH, NY, PNH, and UC. We, therefore, designate the sheet in NY (barcode 00436125) as the lectotype [second-step].

### 32. Rhaphiolepis platyphylla

Taxon classificationPlantaeRosalesRosaceae

(Merr.) B.B.Liu & J.Wen, Front. Plant Sci. 10-1731: 11. 2020.

BBE17A6B-8BAC-5546-930B-6A6B0768DF62

≡ Eriobotryaplatyphylla Merr., Brittonia 4(1): 80. 1941. Type: Myanmar. Upper Burma: hills east of Fort Hertz, 8 December 1931, *F. Kingdon-Ward 10205* (lectotype, designated by Liu et al. 2020c, pg. 109: A [barcode 00026485]!; isolectotypes: A [barcode 00026484]!, BM [barcode BM000602191]!).
≡ Pyrusplatyphylla (Merr.) M.F.Fay & Christenh., Global Fl. 4: 116. 2018. Type: Based on Eriobotryaplatyphylla.

#### Distribution.

Myanmar (Kachin).

### 33. Rhaphiolepis poilanei

Taxon classificationPlantaeRosalesRosaceae

(J.E.Vidal) B.B.Liu & J.Wen, Front. Plant Sci. 10-1731: 11. 2020.

0AC90D38-20ED-5AF0-9D10-0CDFEC5EEECD

≡ Eriobotryapoilanei J.E.Vidal, Adansonia sér. 2, 5: 557. 1965. Type: Vietnam. Haut Donnai: Annam, Canton de Laouan Délégation de Djiriing, alt. 1200 m, 5 June 1933, *E. Poilane 22591* (lectotype, designated by Liu et al. 2020c, pg. 110: P [barcode P02143226]!; isolectotypes: C [barcode C10017885]!, L [barcode L0019414]!, P [barcode P02143227, P02143228]!).
≡ Pyruspoilanei (J.E.Vidal) M.F.Fay & Christenh., Global Fl. 4: 116. 2018. Type: Based on Eriobotryapoilanei.

#### Distribution.

Vietnam (Haut-Donnai).

### 34. Rhaphiolepis prinoides

Taxon classificationPlantaeRosalesRosaceae

(Rehder & E.H.Wilson) B.B.Liu & J.Wen, Front. Plant Sci. 10-1731: 11. 2020.

4098FCCE-6503-5465-B5E9-4C101730A5E5

≡ Eriobotryaprinoides Rehder & E.H.Wilson, Pl. Wilson. (Sargent) 1(2): 194. 1912. Type. China. Yunnan: Mengtze (Mengzi), alt. 1500 m, *A. Henry 9878* (lectotype, designated by Liu et al. 2020c, pg. 110: A [barcode 00026476]!; isolectotypes: A [barcode 00026478]!, B [barcode B 10 0295749]!, E [barcode E00011334]!, K [barcode K000758391, excluding the fruiting branch]!, MO [barcode MO-176739]!, NY [barcode 00436210, excluding the fruiting branch, 00436211, 00436212]!, US [barcode 00097491, excluding the fruiting branch]!).
≡ Pyrusprinoides (Rehder & E.H.Wilson) M.F.Fay & Christenh., Global Fl. 4: 116. 2018. Type: Based on Eriobotryaprinoides.

### 34a. Rhaphiolepis prinoides var. prinoides

Taxon classificationPlantaeRosalesRosaceae

5468A9D6-8C5E-53FA-8207-BAE1D0D070A4

#### Distribution.

China (Sichuan and Yunnan) and Laos.

### 34b. Rhaphiolepis prinoides (Rehder & E.H.Wilson) B.B.Liu & J.Wen var. laotica

Taxon classificationPlantaeRosalesRosaceae

(J.E.Vidal) B.B.Liu & J.Wen, Front. Plant Sci. 10-1731: 11. 2020.

09F0B624-C6C4-5B08-883D-E1D69AB1BF7A

≡ EriobotryaprinoidesRehder & E.H.Wilsonvar.laotica J.E.Vidal, Adansonia sér. 2, 5: 573. 1965. Type. Laos. Xièng Khouang: 1200 m, en fleurs, 3 November 1920, *E. Poilane 2243* (lectotype, designated by Liu et al. 2020c, pg. 110: P [barcode P02143229]!; isolectotypes: P [barcode P02143230, P02143231]!).

#### Distribution.

Laos (Xièng Khouang).

### 35. Rhaphiolepis salicifolia

Taxon classificationPlantaeRosalesRosaceae

Lindl., Coll. Bot. (Lindley) 1: sub t. 3. 1821.

8ABC84F6-B8FB-52A1-A95E-97C3BDE2B4D3

≡ Pyrusgomorrana M.F.Fay & Christenh., Global Fl. 4: 105. 2018. Type: not designated.
= Rhaphiolepischeniana F.P.Metcalf, Lingnan Sci. J. 18: 509. 1939. “Raphiolepis” Type: China. Fujian: Nanputo, Amoy, January 2, 1927, *H.H. Chung 5912* (holotype: A [barcode 00032513]!; isotype: A [barcode 00032514]!).
= Rhaphiolepiskwangsiensis Hu, J. Arnold Arbor. 13: 335. 1932. Type: China. Guangxi, Me-Kom, Seh-feng Dar-Shan, S. Nanning, 800m, *R.C. Ching 8360* (holotype: A [barcode 00032541]!, isotype: A [barcode 00032542]!).

#### Distribution.

China (Fujian, Guangdong, and Guangxi) and Vietnam (Quang Tri and Thua Thiên).

### 36. Rhaphiolepis salwinensis

Taxon classificationPlantaeRosalesRosaceae

(Hand.-Mazz.) B.B.Liu & J.Wen, Front. Plant Sci. 10-1731: 11. 2020.

25E18746-E67A-519B-BF5D-2726934137DE

≡ Eriobotryasalwinensis Hand.-Mazz., Symb. Sin. Pt. 7(3): 475. 1933. Type: China. Yunnan: Im str. Laubwalde des birm. Mons. am Ufer des Salwin um Tschamutong von Sijitong bis unter Tjiontson, Phyllit und kristallinischer Kalk, 1625–1700 m, 13 July & 17 August 1916, *H.F. von Handel-Mazzetti 9573* (lectotype, designated by Liu et al. 2020c, pg. 111: WU [barcode WU0059392]!; isolectotype: A [barcode 00026480]!).
≡ Pyrussalwinensis (Hand.-Mazz.) M.F.Fay & Christenh., Global Fl. 4: 120. 2018. Type: Based on Eriobotryasalwinensis.

#### Distribution.

China (NE Yunnan and Tibet), India, and Myanmar.

### 37. Rhaphiolepis seguinii

Taxon classificationPlantaeRosalesRosaceae

(H.Lév.) B.B.Liu & J.Wen, Front. Plant Sci. 10-1731: 11. 2020.

3A98D93B-EB4D-5107-838C-8EA2A4345E84

≡ Symplocosseguinii H.Lév., Repert. Spec. Nov. Regni Veg. 10: 431. 1912. Type: China. Kouy-Tchéou (Guizhou): Environs de Ou-La-Gay et de Hoang-Ko-Chou, Mars 1899, *J. Séguin & R.P. Bodinier 2617* (lectotype, selected by Vidal 1965, pg. 575, first step “type”; second step, designated by Liu et al. 2020c, pg. 114: E [barcode E00011359]!; isolectotypes: P [barcode P02143232, P02143233]!).
≡ Eriobotryaseguinii (H.Lév.) Cardot ex Guillaumin, Bull. Soc. Bot. France 71: 287, in obs. 1924. Type: Based on Symplocosseguinii.
= Eriobotryapseudorhaphiolepis Cardot, Notul. Syst. (Paris) 3: 371. 1918. nom. nov. superfl. Type: Based on Symplocosseguinii (Referring to the note Liu et al. 2020c, pg. 114).

#### Distribution.

China (SW Guizhou and SE Yunnan).

### 38. Rhaphiolepis serrata

Taxon classificationPlantaeRosalesRosaceae

(J.E.Vidal) B.B.Liu & J.Wen, Front. Plant Sci. 10-1731: 12. 2020.

D648B14B-3B8D-5243-9772-B646B8F8691E

≡ Eriobotryaserrata J.E.Vidal, Adansonia sér. 2, 5: 558. 1965. Type: Laos. Xièng Khouang: Ban Na Poun, 1200 m, en fleurs, 19 November 1920, *E. Poilane 2345* (lectotype, designated by Liu et al. 2020c, pg. 111: P [barcode P02143235]!; isolectotypes: A [barcode 00026486]!, L [barcode L0019415]!, P [barcode P02143236, P02143237]!).
≡ Pyrusserrata (J.E.Vidal) M.F.Fay & Christenh., Global Fl. 4: 121. 2018. Type: Based on Eriobotryaserrata.

#### Distribution.

China (Guangxi and Yunnan) and Laos (Xièng Khouang).

### 39. Rhaphiolepis stipularis

Taxon classificationPlantaeRosalesRosaceae

(Craib) B.B.Liu & J.Wen, Front. Plant Sci. 10-1731: 12. 2020.

FA302679-83C4-59B0-A02D-6167894E95F8

≡ Eriobotryastipularis Craib, Bull. Misc. Inform. Kew 1929(4): 109. 1929. Type: Thailand [Siam]: Satul, Adang, 1500 m, on rocky ridge, 16 January 1928, *A.F.G. Kerr 14125* (lectotype, designated by Liu et al. 2020c, pg. 111: K [barcode K000758408]!; isolectotypes: ABD, BK [barcode BK257292]!, BM, K [barcode K000758408]!, TCD [barcode TCD0016606]!).
≡ Pyrusstipularis (Craib) M.F.Fay & Christenh., Global Fl. 4: 122. 2018. Type: Based on Eriobotryastipularis.

#### Distribution.

Cambodia and Thailand (Satun).

### 40. Rhaphiolepis tengyuehensis

Taxon classificationPlantaeRosalesRosaceae

(W.W.Sm.) B.B.Liu & J.Wen, Front. Plant Sci. 10-1731: 12. 2020.

379D7B28-F8F4-5316-BDBF-917A212F223F

≡ Eriobotryatengyuehensis W.W.Sm., Notes Roy. Bot. Gard. Edinburgh 10: 30. 1917. Type: China. Yunnan: Shweli-Salween divide, Lat. 25°5'N, alt. 7000 ft., tree of 40–60 ft., flowers creamy-yellow, open forests, May 1913, *G. Forest 9857* (lectotype, designated by Vidal 1965, pg. 571: E [barcode E00011333]!).
≡ Pyrustengyuehensis (W.W.Sm.) M.F.Fay & Christenh., Global Fl. 4: 123. 2018. Type: Based on Eriobotryatengyuehensis.

#### Distribution.

China (NW Yunnan and Tibet) and Myanmar (Kachin).

### 41. Rhaphiolepis umbellata

Taxon classificationPlantaeRosalesRosaceae

(Thunb.) Makino, Bot. Mag. (Tokyo) 16: 13. 1902.

8B71B1E6-D536-574B-A875-AA52A563B2F9

≡ Laurusumbellata Thunb., Fl. Jap. (Thunberg) 175. 1784. Type: Japan. *Thunberg s.n.* (holotype: UPS-THUNB accession no. 9844).
≡ Rhaphiolepisumbellata C.K.Schneid., Ill. Handb. Laubholzk. i. 705. 1906. Type: Based on Laurusumbellata.
≡ Rhaphiolepisindica(L.)Lindl. subsp. umbellata (Thunb.) Hatus. 1970. Type: Based on Laurusumbellata.
≡ Rhaphiolepisindica(L.)Lindl.f.umbellata (Thunb.) Hatus., J. Geobot. 25(4): 126. 1978. Type: Based on Laurusumbellata.
≡ Rhaphiolepisindica(L.)Lindl.var.umbellata (Thunb.) H.Ohashi, J. Jap. Bot. 63(1): 4. 1988, pro parte. Type: Based on Laurusumbellata.

### 41a. Rhaphiolepis umbellata var. umbellata

Taxon classificationPlantaeRosalesRosaceae

52230948-6F04-5A3D-A117-662E06C0C6B9

= Mespilussieboldii Blume, Bijdr. Fl. Ned. Ind. 17: 1102. 1826. Type: not designated.
≡ Photiniasieboldii (Blume) G.Don, Gen. Hist. 2: 602. 1832. Type: Based on Mespilussieboldii.
≡ Rhaphiolepissieboldii (Blume) Hassk., Flora 25(2): 47. 1842. Type: Based on Mespilussieboldii.
= Rhaphiolepisjaponica Siebold & Zucc., Fl. Jap. (Siebold) 1: 162. 1841. Type: not designated.
≡ Opajaponica (Siebold & Zucc.) Seem., J. Bot. 1: 281. 1863. Type: Based on Rhaphiolepisjaponica.
= Rhaphiolepisovata Briot, Rev. Hort. [Paris]. 348. 1870–1871. Type: not designated.
≡ Rhaphiolepisumbellata(Thunb.)Makinof.ovata (Briot) C.K. Schneid., Ill. Handb. Laubholzk.1: 706. 1906. Type: Based on Rhaphiolepisovata.
≡ RhaphiolepismertensiiSiebold & Zucc.var.ovata (Briot) Nakai, Fl. Sylv. Kor. 6: 32. 1916. Type: Based on Rhaphiolepisovata.

#### Distribution.

China (Taiwan and Zhejiang), Korea, and Japan.

### 41b. Rhaphiolepis umbellata (Thunb.) Makino var. liukiuensis

Taxon classificationPlantaeRosalesRosaceae

Koidz., J. Coll. Sci. Imp. Univ. Tokyo 34(2): 73. 1913.

1E5D3CBA-4862-58BC-AC47-C1CB8096152C

≡ Rhaphiolepisindica(L.)Lindl. subsp. umbellata(Thunb.) Hatus.var.liukiuensis Koidz., J. Coll. Sci. Imp. Univ. Tokyo 34 (2): 73. 1913. Type: South Korea. Jeju Island: “Quelpaert: in rupibus littoris”, May 1907, *U. Faurie 1562* (**lectotype**, selected by Nakai 1924, pg. 64, first step “type”; **second step, designated here**: P [barcode P02143131]!; isolectotypes: A [barcode 00032546]!, B [barcode B 10 0278052]!). [Note Q]
≡ Rhaphiolepisliukiuensis (Koidz.) Nakai, J. Arnold Arbor. 5: 63. 1924. Type: Based on Rhaphiolepisindica subsp. umbellatavar.liukiuensis.
≡ Rhaphiolepisumbellata(Thunb.)Makinosubsp.liukiuensis (Koidz.) Masam. & Yanagih., Trans. Nat. Hist. Soc. Formosa XXXI: 274. 1941. Type: Based on Rhaphiolepisindica subsp. umbellatavar.liukiuensis.
≡ Rhaphiolepisindica(L.)Lindl.var.liukiuensis (Koidz.) Kitam., Acta Phytotax. Geobot. 26(1–2): 2. 1974. Type: Based on Rhaphiolepisindica subsp. umbellatavar.liukiuensis.

#### Distribution.

China (Taiwan), Japan (Kyushu and Ryukyu), and Korea (Jeju Island).

#### Note Q.

Koidzumi (1913) described Rhaphiolepisumbellatavar.liukiuensis and did not mention any type material except the locality “Liukiu: Okinawasima; Korea: Quelpaert”. Nakai designated the collection *U. Faurie 1562* as type, however, it should be lectotype [first-step] according to Art. 9.17 (Turland et al. 2018). This designation of the lectotype is found to refer to three sheets in herbaria A, B, and P, and needs to be further narrowed to a single one of these specimens by way of a subsequent lectotypification [second-step].

### 42. Rhaphiolepis wardii

Taxon classificationPlantaeRosalesRosaceae

(C.E.C.Fisch.) B.B.Liu & J.Wen, Front. Plant Sci. 10-1731: 12. 2020.

A0A5AE5B-B428-5FD6-88F9-63DB457F31F7

≡ Eriobotryawardii C.E.C.Fisch., Bull. Misc. Inform. Kew 1929(6): 205. 1929. Type: Myanmar. Namkiu Mountains. Valley of the Sheinghku, 6000–7000 ft., in flower in October, *F. Kingdon-Ward 7618* (holotype: K [barcode K000758392]!; isotype: A [barcode 00026488]! image of the holotype with a small fragment of inflorescence).
≡ Pyrusalabaster M.F.Fay & Christenh., Global Fl. 4: 94. Feb. 2018. Type: Based on Eriobotryawardii.
≡ Pleiosorbuswardii (C.E.C.Fisch.) Rushforth, Phytologia 100(4): 233. 21 Dec. 2018. Type: Based on Eriobotryawardii.

#### Distribution.

Myanmar (Kachin and North Triangle).

### 43. Rhaphiolepis williamtelliana

Taxon classificationPlantaeRosalesRosaceae

(M.F.Fay & Christenh.) B.B.Liu & J.Wen, Front. Plant Sci. 10-1731: 12. 2020.

A002AE08-91D1-5FD4-8FD8-1E779D35C8C7

≡ Eriobotryafragrans Champ., Hooker’s J. Bot. Kew Gard. Misc. 4: 80. 1852. Type: China. Hong Kong: Mt. Victoria, *J.G. Champion s.n.* (lectotype, designated by Vidal 1965, pg. 557: K [barcode K000758384]! “type”).
≡ Pyruswilliamtelliana M.F.Fay & Christenh., Global Fl. 4: 126. 2018. Type: Based on Eriobotryafragrans.

### 43a. Rhaphiolepis williamtelliana var. williamtelliana

Taxon classificationPlantaeRosalesRosaceae

A87BCD3B-DA4A-5A55-A38B-657019E04ED2

#### Distribution.

China (Guangdong, Guangxi, Hainan, Hongkong, and Tibet) and Vietnam.

### 43b. Rhaphiolepis williamtelliana (M.F.Fay & Christenh.) B.B.Liu & J.Wen var. furfuracea

Taxon classificationPlantaeRosalesRosaceae

(J.E.Vidal) B.B.Liu & J.Wen, Front. Plant Sci. 10-1731: 12. 2020.

286862F5-A1FC-5AB7-AB24-C6076F23A367

≡ EriobotryafragransChamp. ex Benth.var.furfuracea J.E.Vidal, Adansonia sér. 2, 5: 557. 1965. Type: Vietnam (Sud-Annam). Nha Trang: Massif du Hon Ba, 1000–1500 m, en fleurs, 5 September 1918, *A. Chevalier 38893* (lectotype, designated by Liu et al. 2020c, pg. 106: P [barcode P02143263]!; isolectotypes: A [barcode 00026481]!, C [barcode C10017884]!, K [barcode K000758407]!, L [barcode L0019413]!, P [barcode P02143264, P02143265, P02143266]!).

#### Distribution.

Vietnam (Nha Trang).

### 44. Rhaphiolepis wuzhishanensis

Taxon classificationPlantaeRosalesRosaceae

W.B.Liao, R.H.Miao & Q.Fan, Novon 17(4): 429 (-431, fig. 1). 2007.

C6DC4F92-7B52-5A04-9ADE-0F328CEAFC22

#### Type.

China. Hainan: Mt. Wuzhishan, 1830 m, 5 August 2005, *Q. Fan 6087* (holotype: SYS; isotype: MO).

#### Distribution.

China (Hainan).

### 45. Rhaphiolepis yui

Taxon classificationPlantaeRosalesRosaceae

B.B.Liu & J.Wen, nom. nov. [Note R]

D67EC4FB-138C-5608-A82A-670B69806CB2

urn:lsid:ipni.org:names:77210703-1

≡ Eriobotryakwangsiensis Chun ex X.H.Yang & S.Q.Lin, Acta Hortic. 750: 221. 2007. Type: China. Guangxi: Xiangzhou County, Shangguchen, Wuzhishan, 18 June 1936, *C. Wang 39423* (holotype: IBK [barcode IBK00061038, herbarium accession number 35925]!; isotypes: PE [barcode 00799311]!, SZ [barcode 00194327]). [Note S]

#### Distribution.

China (Guangxi).

#### Note R.

The species epithet “kwangsiensis” has been pre-occupied by *Rhaphiolepiskwangsiensis* Hu, J. Arnold Arbor. 13: 335. 1932, thus a new name is needed for this taxon (Turland et al. 2018). The epithet is given in honor of the late Prof. Te-Tsun Yu (PE) for his great contributions to the taxonomy of Rosaceae.

#### Note S.

**Figure 2.**
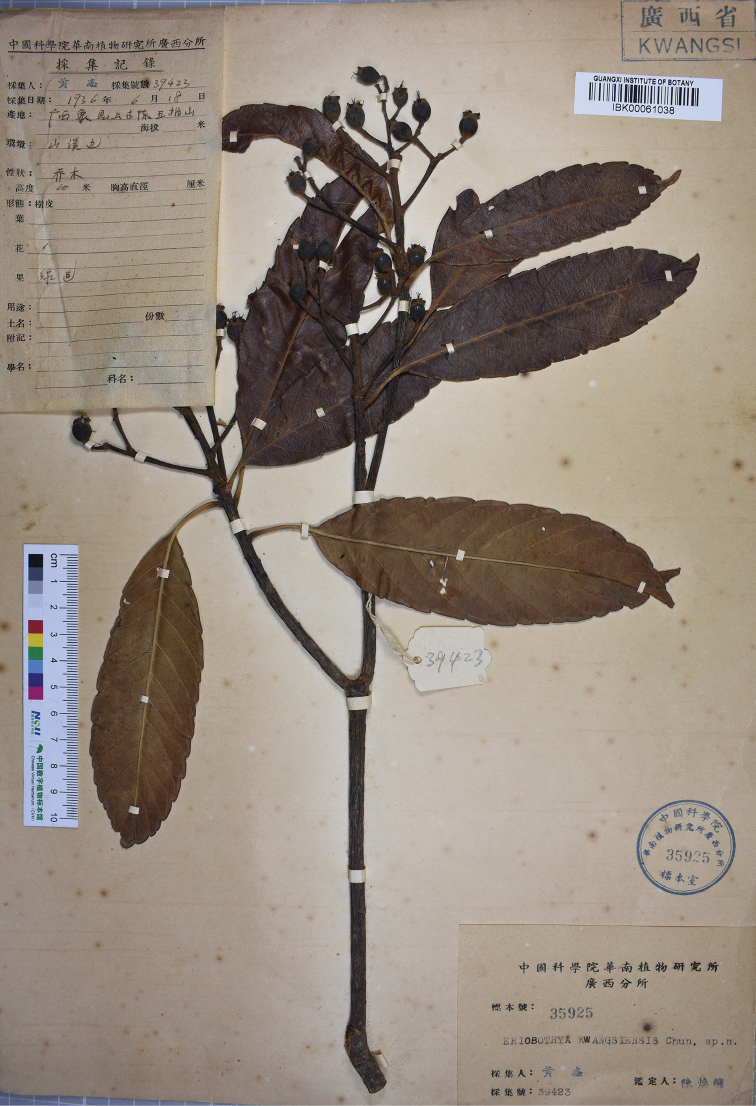
Holotype of *Rhaphiolepisyui* (IBK [barcode IBK00061038]).

Yang and Lin (2007) provided the following type information in the protologue, “Guangxi: Shangguchen. 1936. 6. *C. Wang 35925* (Holotypus, TBK)”. According to the image of holotype provided in the protologue, the holotype is *C. Wang 39423*, which was deposited in the herbarium IBK rather than TBK (Fig. 2). The number “35925” cited in Yang and Lin (2007) was the herbarium accession number of the holotype.

**Artificial hybrid species**:

**Rhaphiolepis×delacourii André, Rev. Hort. [Paris]. 72: 698. 1900.**

≡ *Pyrus×delacourii* (André) M.F.Fay & Christenh., Global Fl. 4: 101. 2018. Type: France. Golfe-Juan, May 1900, *s.coll. s.n.* (holotype: K [barcode K000758248]!). [Note T]

**Note T.** This hybrid species was produced by M. Delacour, a gardener of the Villa Allerton, in Cannes, and it was hybridized between *Rhaphiolepisindica* and *R.ovata* (= *R.umbellata*).

**Doubtful names associated with *Rhaphiolepis*** :

PhotinialuzonensisMerr.var.acuminatissima Merr., nom. nud.

*Rhaphiolepiscrassifolia* Hort. ex Voll & Brade, Rodriguésia i. No. 3, 61. 1935, nom. nud.

*Rhaphiolepishiiranensis* Kaneh., Formosan Trees, ed. rev. 276. 1936, anglice et japonice. ≡ Rhaphiolepisindica(L.)Lindl.var.hiiranensis (Kaneh.) H.L.Li, Lloydia 4: 235. 1952. ≡ Rhaphiolepisumbellata(Thunb.)Makinovar.hiiranensis (Kaneh.) Hatus., Fl. Ryukyus 312. 1971. nom. nud.

Rhaphiolepisindica(L.)Lindl.var.insularis Hatus., Fl. Ryukyus 844. 1971. nom. illeg. & nom. nud.

*Rhaphiolepislaevis* Lodd. ex G.Don, Hort. Brit. [Loudon] 202. 1830, “*Raphiolepis*”. nom. nud.

*Rhaphiolepislatifolia* Lodd. ex G.Don, Hort. Brit. [Loudon] 202. 1830, “*Raphiolepis*”. nom. nud.

*Rhaphiolepispheostemonia* St.-Lag., Ann. Soc. Bot. Lyon vii. 133. 1880. nom. nud.

## Supplementary Material

XML Treatment for Rhaphiolepis

XML Treatment for Rhaphiolepis angustissima

XML Treatment for Rhaphiolepis balgooyi

XML Treatment for Rhaphiolepis bengalensis

XML Treatment for Rhaphiolepis bengalensis f. bengalensis

XML Treatment for Rhaphiolepis bengalensis (Roxb.) B.B.Liu & J.Wen f. angustifolia

XML Treatment for Rhaphiolepis bengalensis (Roxb.) B.B.Liu & J.Wen f. contracta

XML Treatment for Rhaphiolepis bengalensis (Roxb.) B.B.Liu & J.Wen f. gigantea

XML Treatment for Rhaphiolepis bengalensis (Roxb.) B.B.Liu & J.Wen f. intermedia

XML Treatment for Rhaphiolepis bengalensis (Roxb.) B.B.Liu & J.Wen f. multinervata

XML Treatment for Rhaphiolepis bibas

XML Treatment for Rhaphiolepis brevipetiolata

XML Treatment for Rhaphiolepis cavaleriei

XML Treatment for Rhaphiolepis condaoensis

XML Treatment for Rhaphiolepis × daduheensis

XML Treatment for Rhaphiolepis deflexa

XML Treatment for Rhaphiolepis dubia

XML Treatment for Rhaphiolepis elliptica

XML Treatment for Rhaphiolepis elliptica var. elliptica

XML Treatment for Rhaphiolepis elliptica (Lindl.) B.B.Liu & J.Wen var. petelotii

XML Treatment for Rhaphiolepis ferruginea

XML Treatment for Rhaphiolepis ferruginea var. ferruginea

XML Treatment for Rhaphiolepis ferruginea F.P.Metcalf var. serrata

XML Treatment for Rhaphiolepis fulvicoma

XML Treatment for Rhaphiolepis glabrescens

XML Treatment for Rhaphiolepis glabrescens var. glabrescens

XML Treatment for Rhaphiolepis glabrescens (J.E.Vidal) B.B.Liu & J.Wen var. victoriensis

XML Treatment for Rhaphiolepis henryi

XML Treatment for Rhaphiolepis hookeriana

XML Treatment for Rhaphiolepis indica

XML Treatment for Rhaphiolepis indica var. indica

XML Treatment for Rhaphiolepis indica (L.) Lindl. var. phaeostemon

XML Treatment for Rhaphiolepis indica (L.) Lindl. var. shilanensis

XML Treatment for Rhaphiolepis indica (L.) Lindl. var. spiralis

XML Treatment for Rhaphiolepis indica (L.) Lindl. var. tashiroi

XML Treatment for Rhaphiolepis indica (L.) Lindl. f. impressivena

XML Treatment for Rhaphiolepis indica (L.) Lindl. f. minor

XML Treatment for Rhaphiolepis integerrima

XML Treatment for Rhaphiolepis jiulongjiangensis

XML Treatment for Rhaphiolepis lanceolata

XML Treatment for Rhaphiolepis laoshanica

XML Treatment for Rhaphiolepis latifolia

XML Treatment for Rhaphiolepis longifolia

XML Treatment for Rhaphiolepis macrocarpa

XML Treatment for Rhaphiolepis major

XML Treatment for Rhaphiolepis malipoensis

XML Treatment for Rhaphiolepis merguiensis

XML Treatment for Rhaphiolepis oblongifolia

XML Treatment for Rhaphiolepis obovata

XML Treatment for Rhaphiolepis petiolata

XML Treatment for Rhaphiolepis philippinensis

XML Treatment for Rhaphiolepis platyphylla

XML Treatment for Rhaphiolepis poilanei

XML Treatment for Rhaphiolepis prinoides

XML Treatment for Rhaphiolepis prinoides var. prinoides

XML Treatment for Rhaphiolepis prinoides (Rehder & E.H.Wilson) B.B.Liu & J.Wen var. laotica

XML Treatment for Rhaphiolepis salicifolia

XML Treatment for Rhaphiolepis salwinensis

XML Treatment for Rhaphiolepis seguinii

XML Treatment for Rhaphiolepis serrata

XML Treatment for Rhaphiolepis stipularis

XML Treatment for Rhaphiolepis tengyuehensis

XML Treatment for Rhaphiolepis umbellata

XML Treatment for Rhaphiolepis umbellata var. umbellata

XML Treatment for Rhaphiolepis umbellata (Thunb.) Makino var. liukiuensis

XML Treatment for Rhaphiolepis wardii

XML Treatment for Rhaphiolepis williamtelliana

XML Treatment for Rhaphiolepis williamtelliana var. williamtelliana

XML Treatment for Rhaphiolepis williamtelliana (M.F.Fay & Christenh.) B.B.Liu & J.Wen var. furfuracea

XML Treatment for Rhaphiolepis wuzhishanensis

XML Treatment for Rhaphiolepis yui

## Footnotes

A forma typica differt inflorescentiis contractis, multo brevioribus (Vidal 1965).
